# Supplementary material for: Dynamic rotation of the protruding domain enhances the infectivity of norovirus
Source: PLoS Pathog. 2020 Jul 2;16(7):e1008619. doi: 10.1371/journal.ppat.1008619 (PMC7331980; doi:10.1371/journal.ppat.1008619)
Supplement: S1 Table — (DOCX) [file ppat.1008619.s001.docx]

| Data Collection | |
| --- | --- |
| Electron microscopy | Titan Krios |
| Camera | Falcon II camera |
| Voltage | 300 kV |
| Magnification | 75,000 |
| Calculated pixel size | 0.86 Å |
| Exposure time | 2 s |
| Electron dose | 40 electron/Å^2^ |
| Number of frames | 32 |
| Defocus range | 1.0 - 2.5 μm |
| Image Processing | |
| Frame alignment | MotionCor2 |
| CTF estimation software | CTFFIND 4.1.5 |
| Number of micrographs | 2,746 |
| 3D map reconstruction software | Relion 2.0 |
| Initial number of particles | 44,626 |
| Particles contributing to final map | 41,847 |
| Applied symmetry | I1 |
| Applied B-factor | -157 Å^2^ |
| Global resolution (FSC = 0.143) | 3.53Å |
| EMDB number | EMD-9741 |
| Model Building | |
| Modeling software | Coot, Phenix |
| Number of residues built | 513 (A), 516 (B), 502 (C) |
| R.m.s. deviation (bonds) | 0.007 |
| R.m.s. deviation (angles) | 1.192 |
| Ramachandran outliers | 0 % |
| Rotamer outliers | 0.47 % |
| Clash score, all atoms | 7.97 |
| PDB ID | 6IUK |

**Table S1:**

**Data collection, image processing and model statistics (MNoV-S7 VLP)**
