# Supplementary material for: Dynamic rotation of the protruding domain enhances the infectivity of norovirus
Source: PLoS Pathog. 2020 Jul 2;16(7):e1008619. doi: 10.1371/journal.ppat.1008619 (PMC7331980; doi:10.1371/journal.ppat.1008619)
Supplement: S2 Table — (DOCX) [file ppat.1008619.s002.docx]

**Table S2:**

**Data collection and image processing (MNoV infectious particles , HNoV GII.3 VLP)**

| Data Collection | | |
| --- | --- | --- |
| Electron microscopy | JEM-2200FS | |
| Camera | DE20 | |
| Voltage | 200 kV | |
| Magnification | 40,000 | |
| Calculated pixel size | 1.422 Å | |
| Exposure time | 3 s | |
| Electron dose | 15 electron/Å^2^ | |
| Number of frames | 75 | |
| Defocus range | 2.0 - 4.0 μm | |
| Image Processing | | |
| Frame alignment | Script provided by DE manufacturer | |
| CTF estimation software | CTFFIND 4.1.5 | |
| Number of micrographs | 1,046 ^*1^ | 2,188 ^*2^ |
|  | 2,049 ^*3^ | 2,739 ^*4^ |
|  | 106 ^*5^ | 1,917 ^*6^ |
| Reconstruction software | Relion 2.0 | |
| Initial number of particles | 7,372 ^*1^ | 20,590 ^*2^ |
|  | 18,706 ^*3^ | 17,820 ^*4^ |
|  | 560 ^*5^ | 5,097 ^*6^ |
| Particles contributing to final map | 6,708 ^*1^ | 4,704 ^*2^ |
|  | 17,820 ^*3^ | 5,063 ^*4^ |
|  | 279 ^*5^ | 1,482 ^*6^ |
| Applied symmetry | I1 | |
| Applied B-factor | -371 Å^2 *1^ | -695 Å^2 *2^ |
|  | - 400 Å^2 *3^ | -612 Å^2 *4^ |
|  | - 1000Å^2 *5^ | -1900 Å^2 *6^ |
| Global resolution  (FSC = 0.143) | 5.3 Å ^*1^ | 7.3 Å ^*2^ |
|  | 5.2 Å ^*3^ | 7.2 Å ^*4^ |
|  | 9.3 Å ^*5^ | 13 Å ^*6^ |
| EMDB number | EMD-9737 ^*1^ | EMD-9738 ^*2^ |
|  | EMD-9735 ^*3^ | EMD-9736 ^*4^ |
|  | EMD-9739 ^*5^ | EMD-9740 ^*6^ |

*1 MNoV-1 with the resting P domain conformation,

*2 MNoV-1 with the rising P domain conformation,

*3 MNoV-S7 with the resting P domain conformation,

*4 MNoV-S7 with the rising P domain conformation,

*5 HNoV GII.3 VLP with the resting P domain conformation,

*6 HNoV GII.3 VLP with the rising P domain conformation
