# Supplementary figures and images for: Dynamic rotation of the protruding domain enhances the infectivity of norovirus
Source: PLoS Pathog. 2020 Jul 2;16(7):e1008619. doi: 10.1371/journal.ppat.1008619 (PMC7331980; doi:10.1371/journal.ppat.1008619)

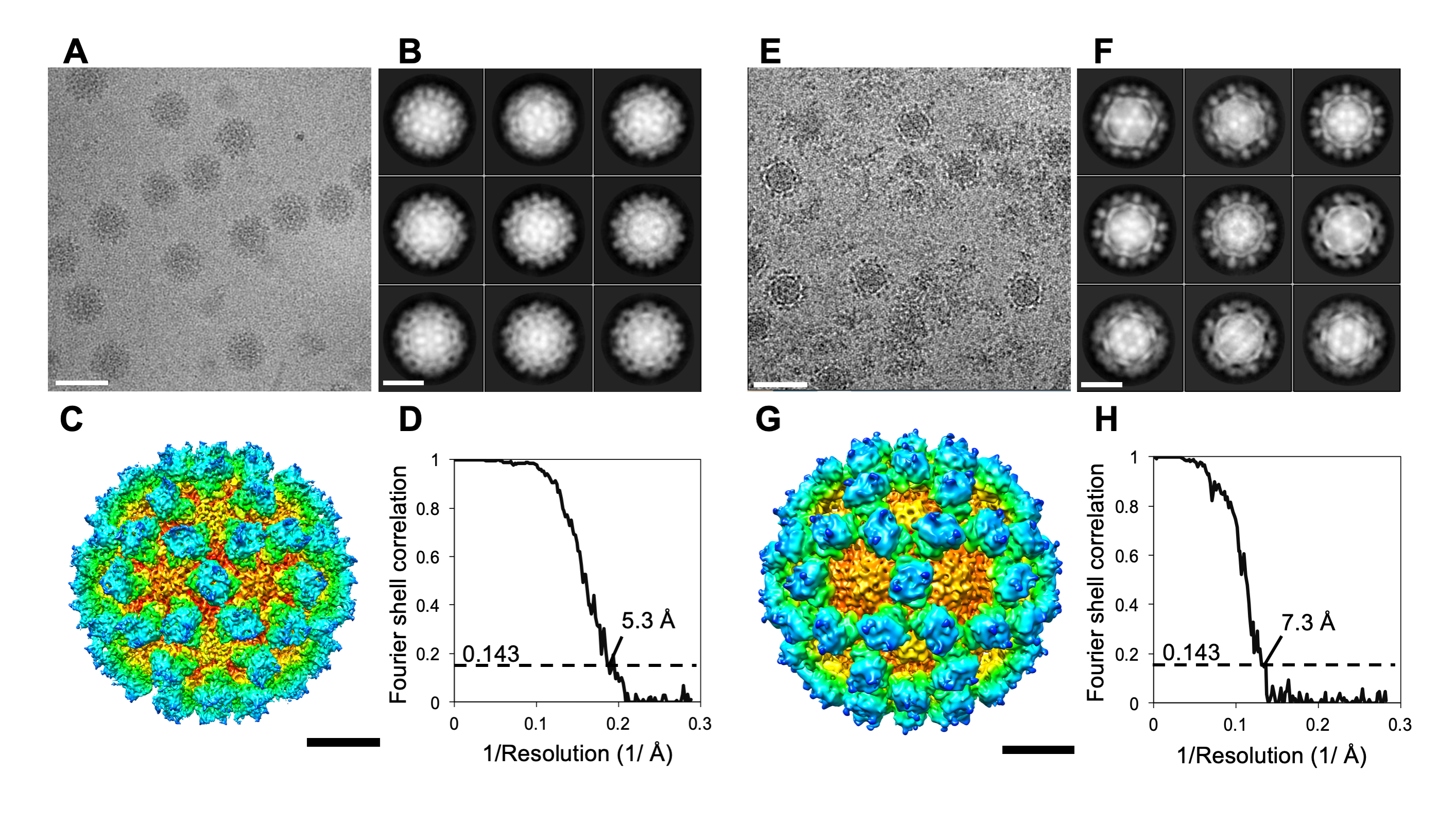

Supplement: S1 Fig — (A and E) Representative micrographs of MNoV-1 in DMEM and PBS(-)+EDTA (pH 8). Scale bars, 500 Å. (B and F) The representative 2D class average images derived from A and E, respectively. Scale bars, 200 Å. (C and G) The surface-shaded depth-cued representations of the MNoV-1 in DMEM and PBS(-)+EDTA (pH 8) viewed along the icosahedral twofold axis. Coloring is based on radii, as in Fig 1A. Scale bars, 100 Å. (D and H) GS-FSC plots of the cryo-EM maps of MNoV-1. Based on the 0.143 criterion for comparing two independent data sets, the image resolutions are estimated to be 5.3 and 7.3 Å, respectively. (TIF) [file ppat.1008619.s003.tif]

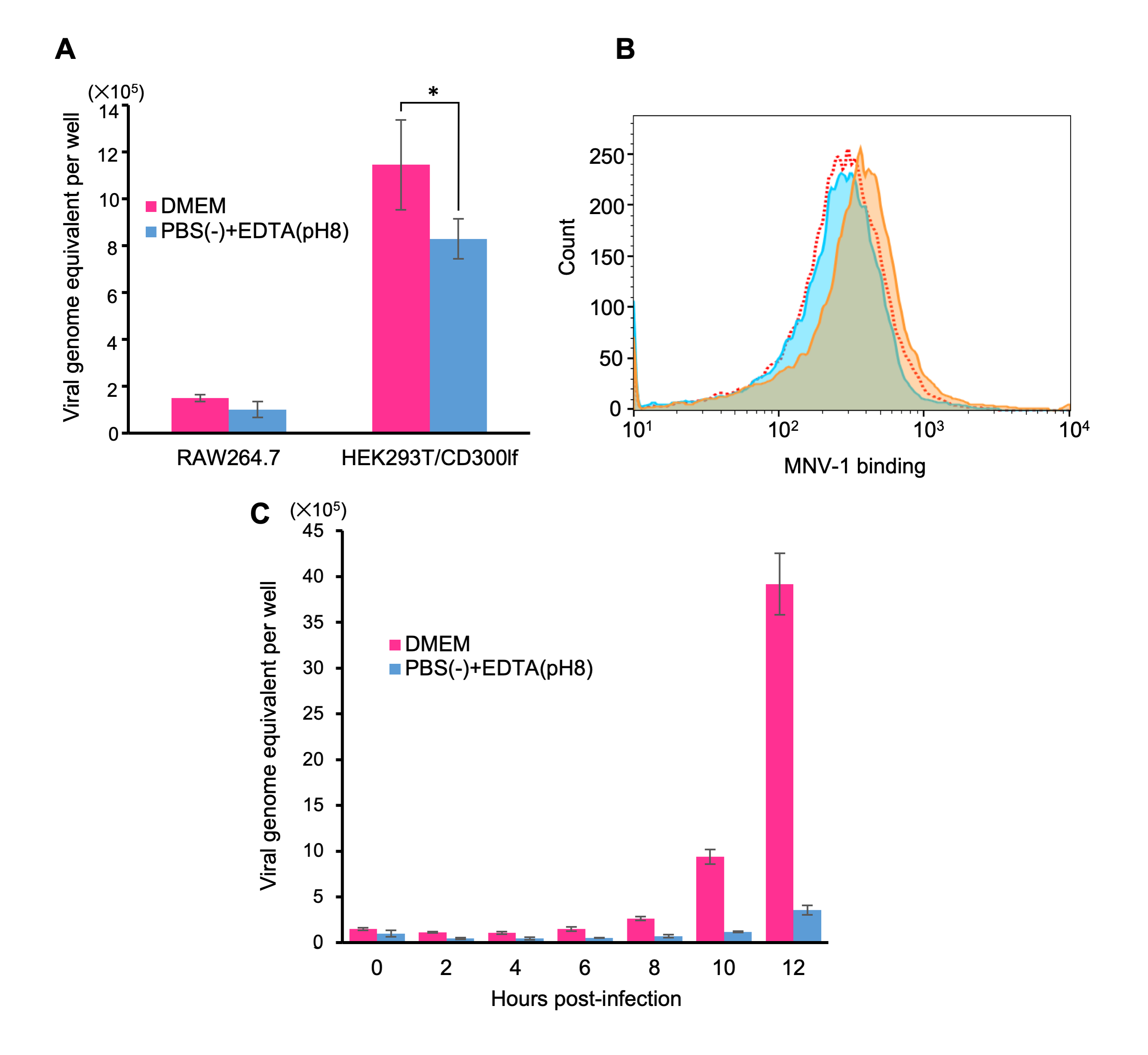

Supplement: S2 Fig — (A) Viral initial attachment on the host cells (RAW264.7 or HEK293T/CD300lf) at 30 minutes after post-infection of the MNoV-1 particles pretreated with DMEM or PBS(-)+EDTA (pH 8). After washing the cells with the solution, the viral RNA was extracted from viruses attached on the cells and the amount was estimated by qRT-PCR. *(p<0.05). (B) The HEK293T/CD300lf cells were incubated with the MNoV-1 particles pretreated with DMEM (Orange), the MNoV-1 particles with PBS(-)+EDTA (pH 8) (Light blue), or without MNoV-1 particles (Red dots). MOI was 10. After incubation, these cells were immunostained with anti-MNoV VP1 antibody. X-axis represents the range of the MNoV binding, and y-axis represents the number of the cells. Median and CV values are 357 and 275 (MNoV-1 particles pretreated with DMEM), and 268 and 157 (MNoV-1 particles pretreated with PBS(-)+EDTA (pH 8)), respectively. (C) Early genome replication of MNoV-1 from 30 minutes to 12 hours. RAW264.7 cells infected with the MNoV-1 particles pretreated with DMEM or PBS(-)+EDTA (pH 8) were washed and collected at each time point of post-infection. The RNA was extracted and the amount was estimated by qRT-PCR. (TIF) [file ppat.1008619.s004.tif]

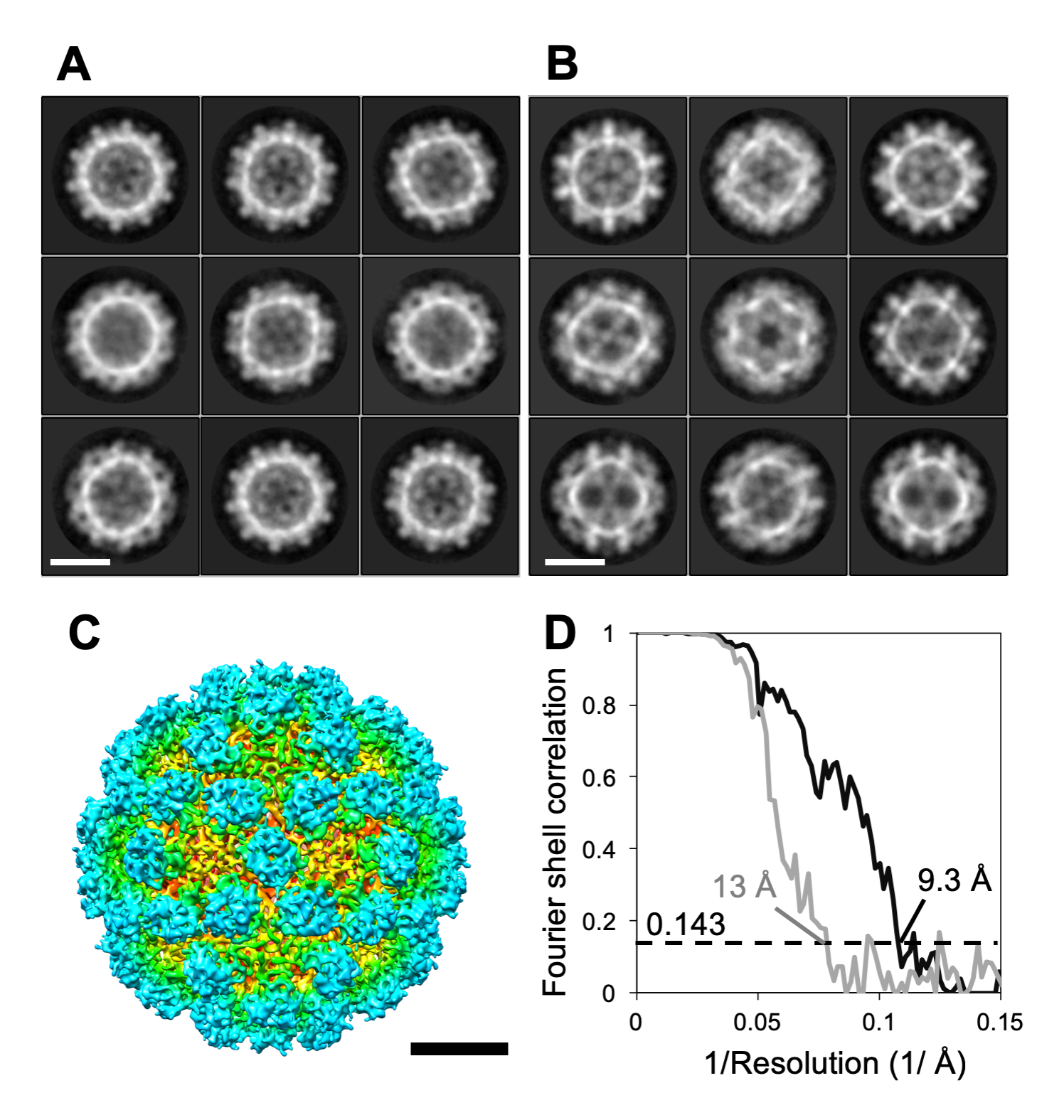

Supplement: S3 Fig — (A and B) Representative 2D class average images of the HNoV GII.3 VLPs with the resting and rising P domain conformations. Scale bars, 200 Å. (C) A surface-shaded depth-cued representation of the HNoV GII.3 VLP with the resting P domain at 9.3 Å resolution. Viewed down the icosahedral twofold axis. Coloring is based on radii as in Fig 1A. Scale bar, 100 Å. (D) GS-FSC plots of the cryo-EM maps of the HNoV GII.3 VLP with the resting and rising P domain conformations indicated by black and gray lines. Based on the 0.143 criterion for comparing two independent data sets, the resolutions of the reconstruction are 9.3 and 13 Å, respectively. (TIF) [file ppat.1008619.s005.tif]

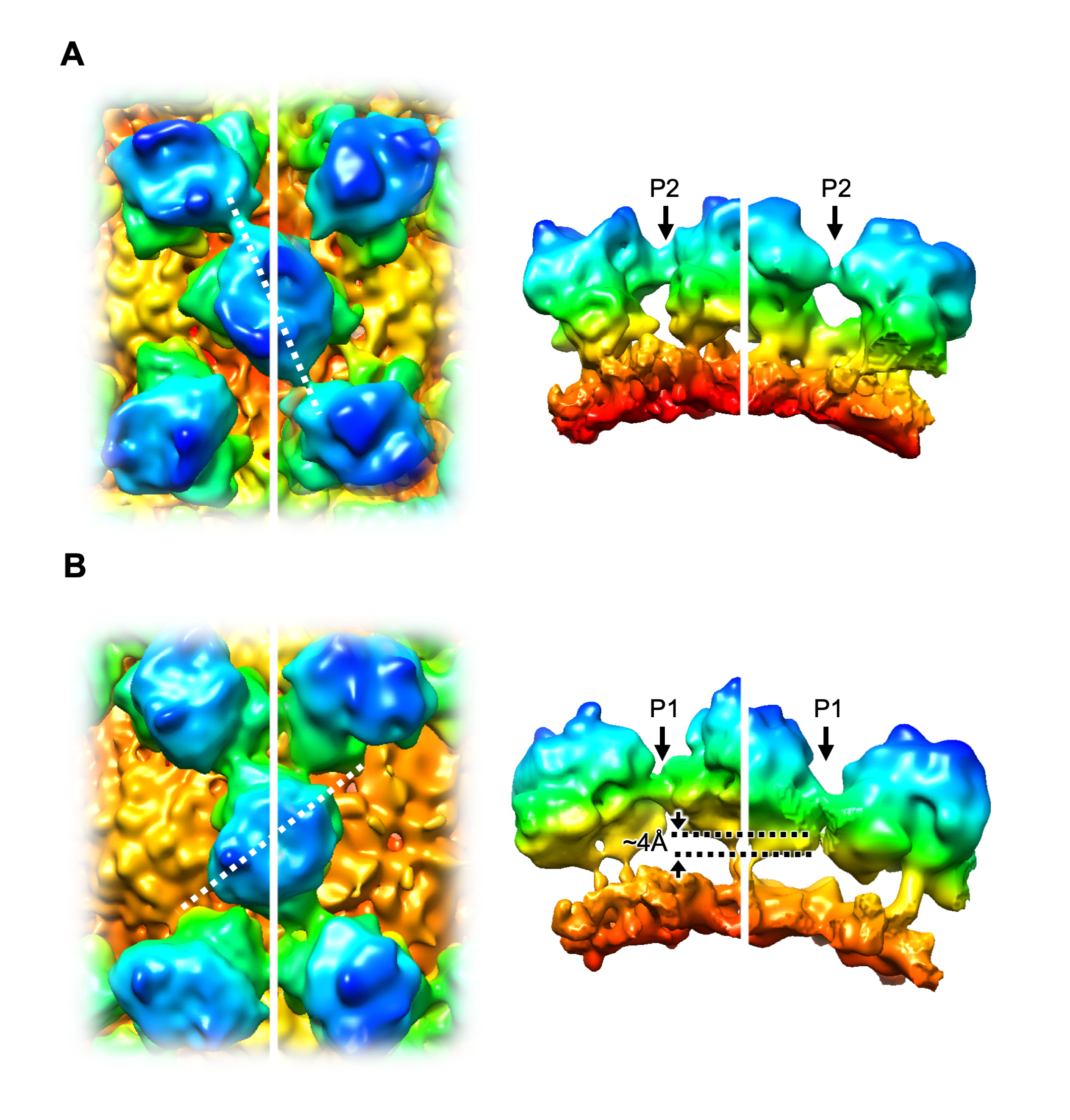

Supplement: S4 Fig — (A) Images of Fig 1C (MNoV-1 with the resting P domain conformation) and Fig 1I (HNoV GII.3 with the resting P domain conformation) are compared side by side. (B) Images of Fig 1D (MNoV-1 with the resting P domain conformation) and Fig 1J (HNoV GII.3 with the resting P domain conformation) are compared side by side. Interaction points between C/C dimer and A/B dimers are labeled by black arrows. In the both strains, the rotation angles between the resting and rising P domains are ~70° clockwise, and the interactions between P domains were also observed similarly at the P2 levels in the resting conformation and at the P1 levels in the rising conformation. However, the heights of the P domain are significantly different in the rising P domain conformations, where the height of P the domain in HNoV GII.3 is ~4 Å shorter than MNoV-1. The P domain structure of MNoV-S7 was similar to that of MNoV-1 at this resolution (in main text). (TIF) [file ppat.1008619.s006.tif]

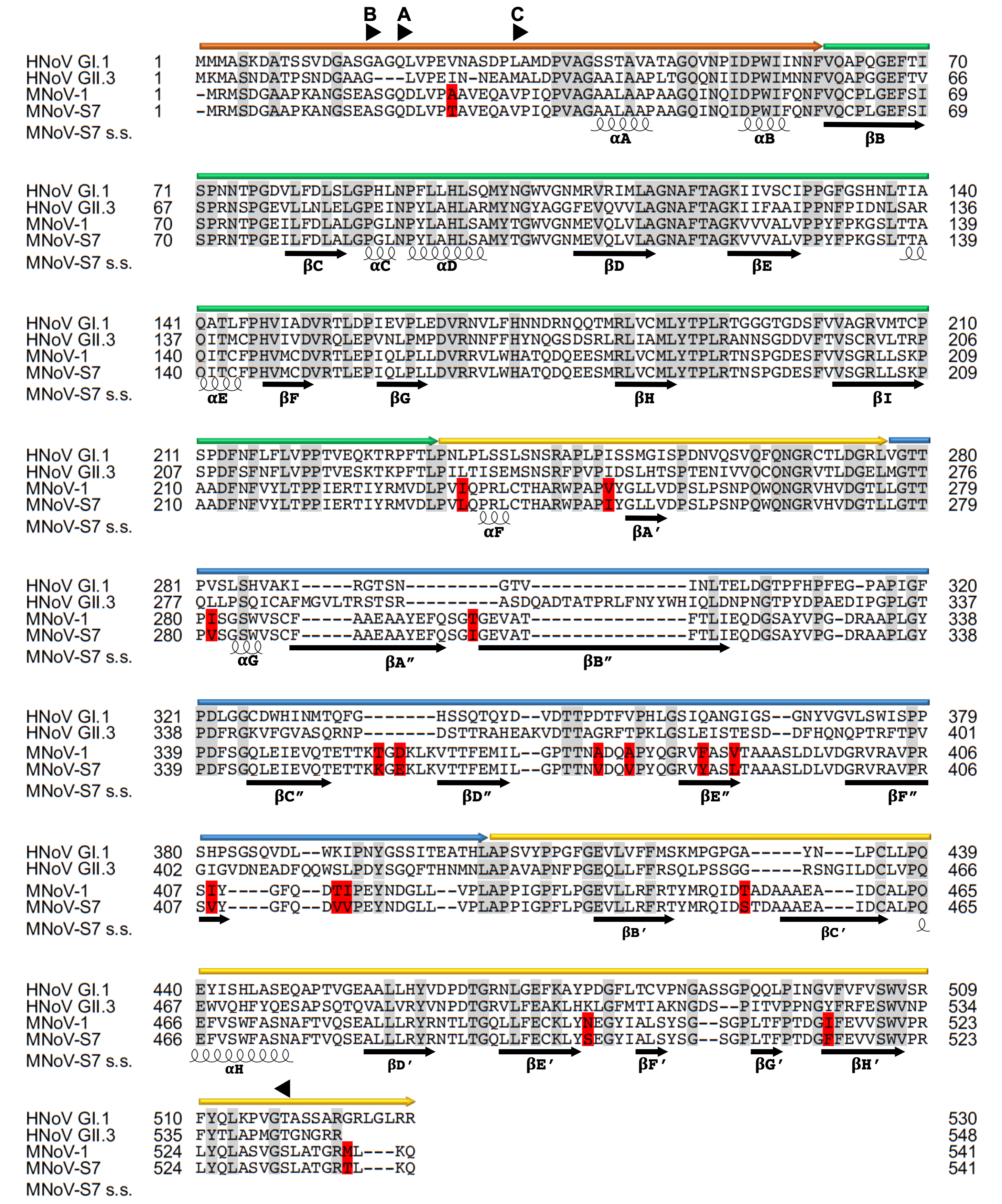

Supplement: S5 Fig — For comparison, the amino acid sequences of the VP1 of HNoV(GI.1, GII.3) and MNoV-1 are aligned to that of MNoV-S7. Crystallographic structures of the HNoV GI.1 S (PDB ID: 1IHM) and the MNoV-1 P (3LQE) domains were used for the initial homology model building of MNoV-S7. Orange, green, yellow, and blue arrows above the alignments represent the N-terminal, S domain, P1 subdomain, and P2 subdomain regions, respectively. Conserved sequences (46%) among HNoV and MNoV are indicated by gray, and different sequences (6%) between MNoV-1 and MNoV-S7 are indicated by red. Regions corresponding to the major β-strands in norovirus capsid are represented by black arrows. Arrowheads indicate start and end residues of the A, B, and C monomers to be built MNoV-S7 structure model, respectively. MNoV-S7 s.s. indicates the predicted secondary structures. (TIF) [file ppat.1008619.s007.tif]

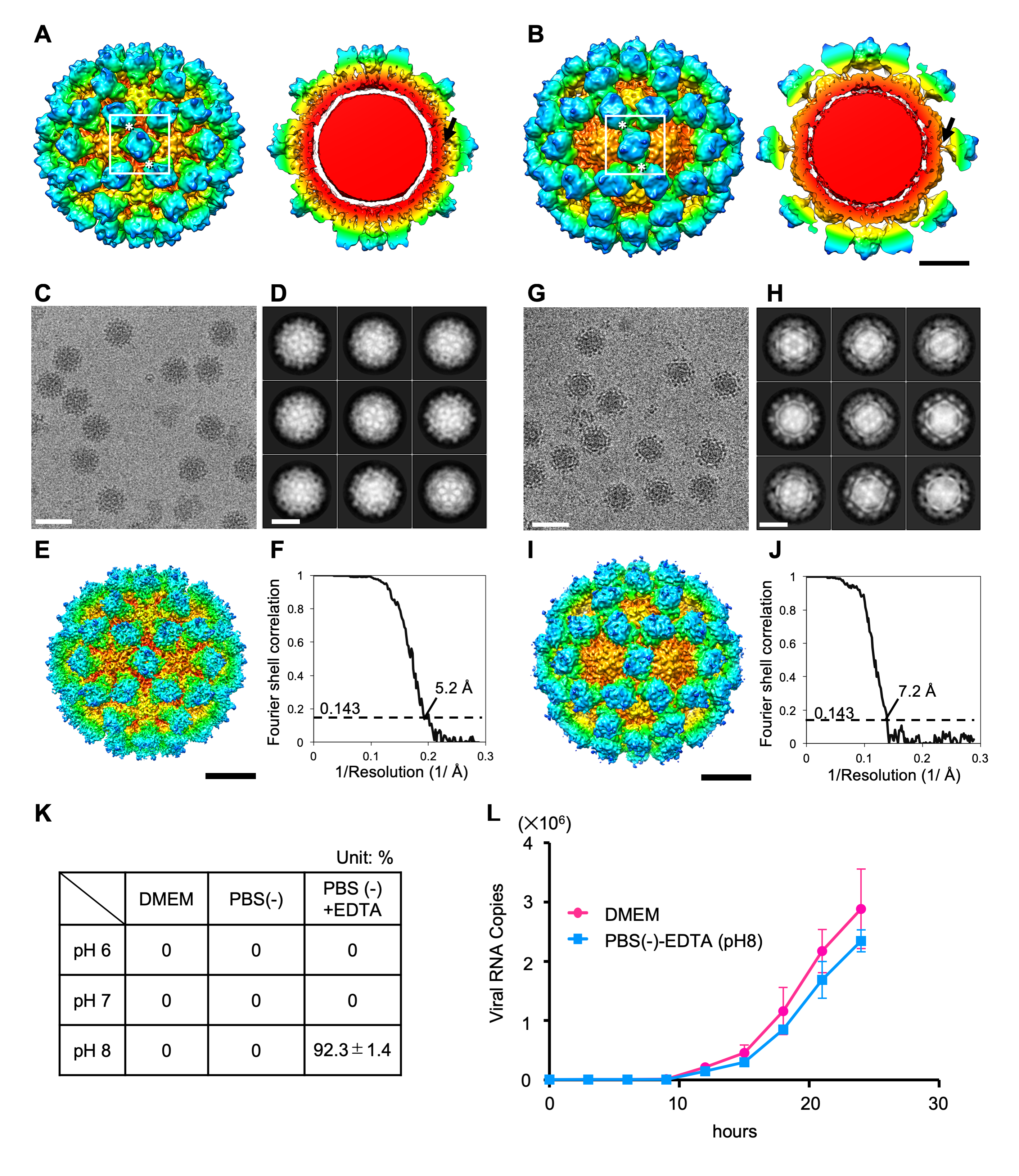

Supplement: S6 Fig — (A and B) Cryo-EM structures of the MNoV-S7 infectious particles, suspended in DMEM and PBS(-)+EDTA (pH 8) are low pass filtered to 8 Å resolution to highlight the P domain structure. Left and right panels show the isosurface display and center section, respectively. The P domain of MNoV-S7 suspended in DMEM rests on the S domain (arrow in A) and interacts with the adjacent P domains at the outer P2 subdomain level (asterisks in A), called the resting P domain conformation. By contrast, the P domain of MNoV-S7 suspended in PBS(-)+EDTA (pH 8) rises off the S domain (arrow in B) and interacts with the adjacent P domains at the inner P1 subdomain level (asterisks in B), called the rising P domain conformation. In the rising P domain conformation, all P domain dimers rotate ~70° clockwise, compared to the resting P domain conformation (highlighted by white boxes), and the interaction sites are changed from the P2 to the P1 levels. The P domain rising off from the S domain in PBS(-)+EDTA (pH 8) reversely rests on the S domain in DMEM. Scale bar, 100 Å. (C and G) Representative micrographs of MNoV-S7 in DMEM and PBS(-)+EDTA (pH 8). Scale bars, 500 Å. (D and H) The representative 2D class average images derived from C and G. Scale bars, 200Å. (E and I) The surface-shaded depth-cued representations of MNoV-S7 in DMEM and PBS(-)+EDTA (pH 8) viewed down the icosahedral twofold axis, respectively. Coloring is based on radii, as in Fig 1A. Scale bar 100 Å. (F and J) GS-FSC Plots of the cryo-EM maps of MNoV-S7 (E and I). Based on the 0.143 criterion for comparing two independent data sets, the resolutions of the reconstruction are 5.2 and 7.3 Å, respectively. (K) The fraction of MNoV-S7 particles exhibiting the rising P domain conformation in different aqueous conditions. Random sampling of 1,000 particles was carried out five times under each condition. The number of particles in each conformation was counted from the individual 2D classes. The rising P domain conformation of M [file ppat.1008619.s008.tif]

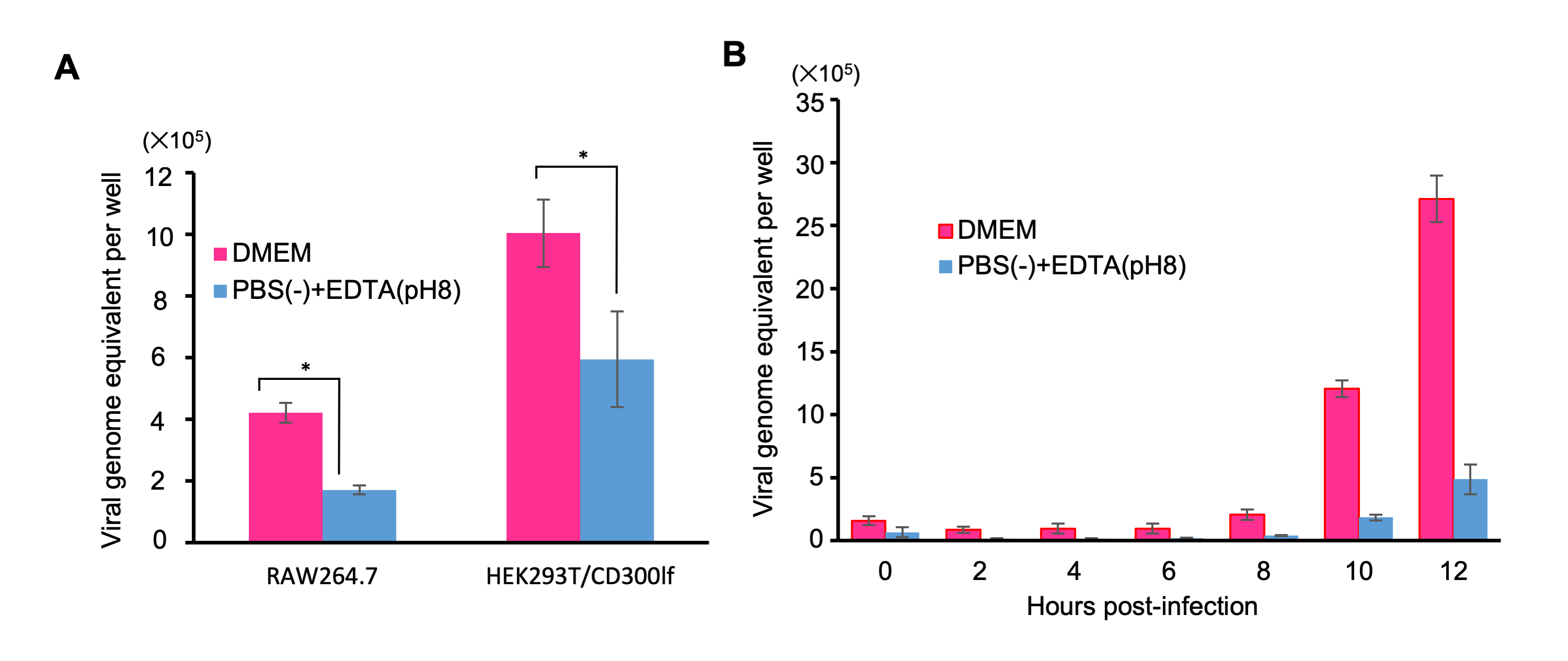

Supplement: S7 Fig — (A) Viral initial attachment on the host cells (RAW264.7 or HEK293T/CD300lf) at 30 minutes after post-infection of the MNoV-S7 particles pretreated with DMEM or PBS(-)+EDTA (pH 8). After washing cells with the solution, the viral RNA was extracted from viruses attached on the cells and the amount was estimated by qRT-PCR. *(p<0.05). (B) Early genome replication of MNoV-S7 from 30 minutes to 12 hours. RAW264.7 cells infected with the MNoV-S7 particles pretreated with DMEM or PBS(-)+EDTA (pH 8) were washed and collected at each time point of post-infection. RNA was extracted and the amount was estimated by qRT-PCR. (TIF) [file ppat.1008619.s009.tif]

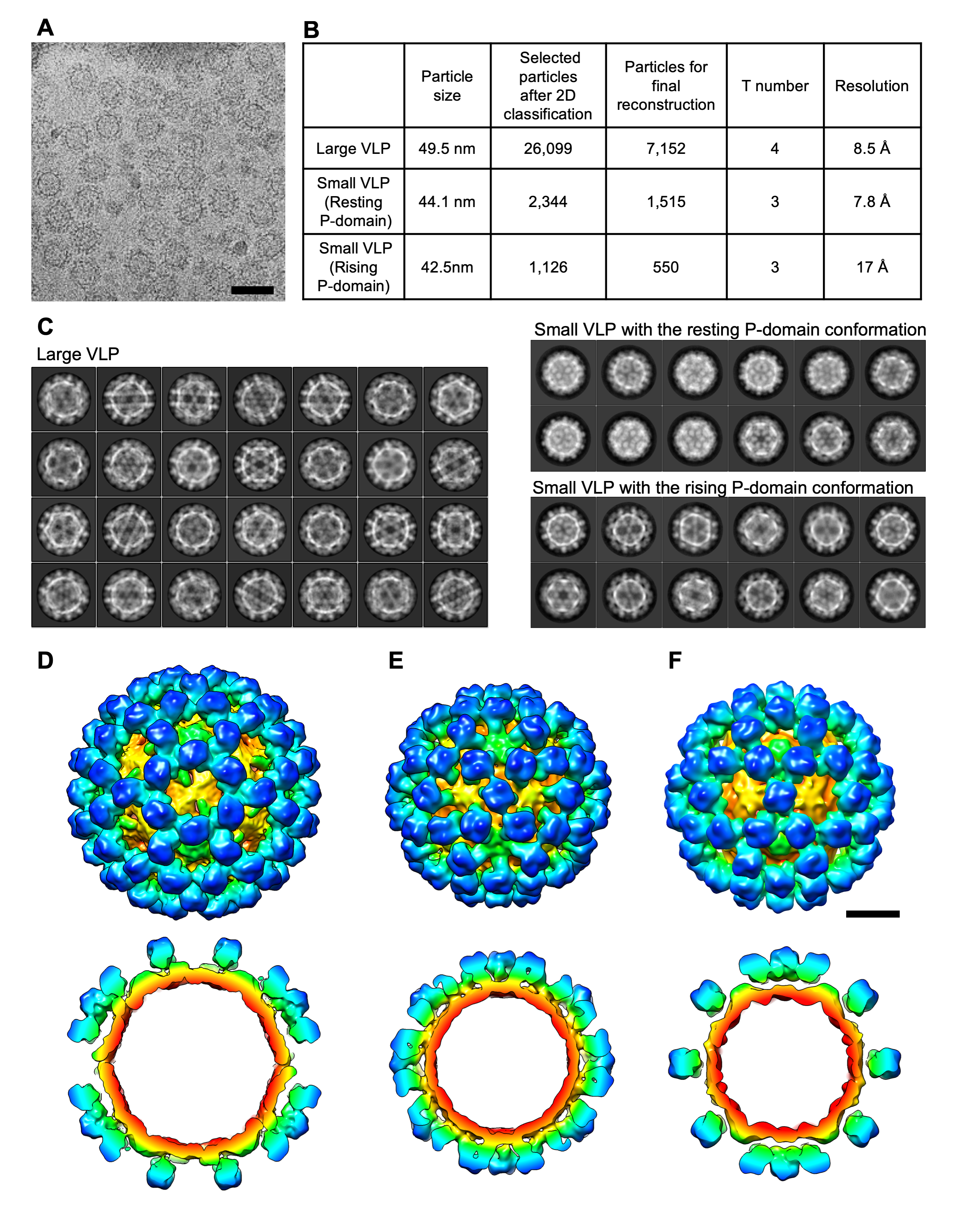

Supplement: S8 Fig — (A) A representative micrograph of the MNoV-1 VLPs. Scale bar, 500 Å. (B) MNoV-1 VLPs contain 3 types of particles; one large particle (T = 4), two small particles (T = 3) with the resting and rising P domain conformations. 3 types of particles were individually subjected to single particle analysis using RELION software. Table shows the number of selected parts after 2D classification, the number of particles used for final reconstruction, T-numbers, and resolutions in 3 types of the particles. (C) Representative 2D class average images in 3 types of particles. Scale bar, 200 Å. (D-F) 3D reconstructions of 3 types of the MNoV-1 VLPs; large particle (D), small particles with the resting (E) and rising (F) P domain conformations. Scale bar, 100 Å. (TIF) [file ppat.1008619.s010.tif]

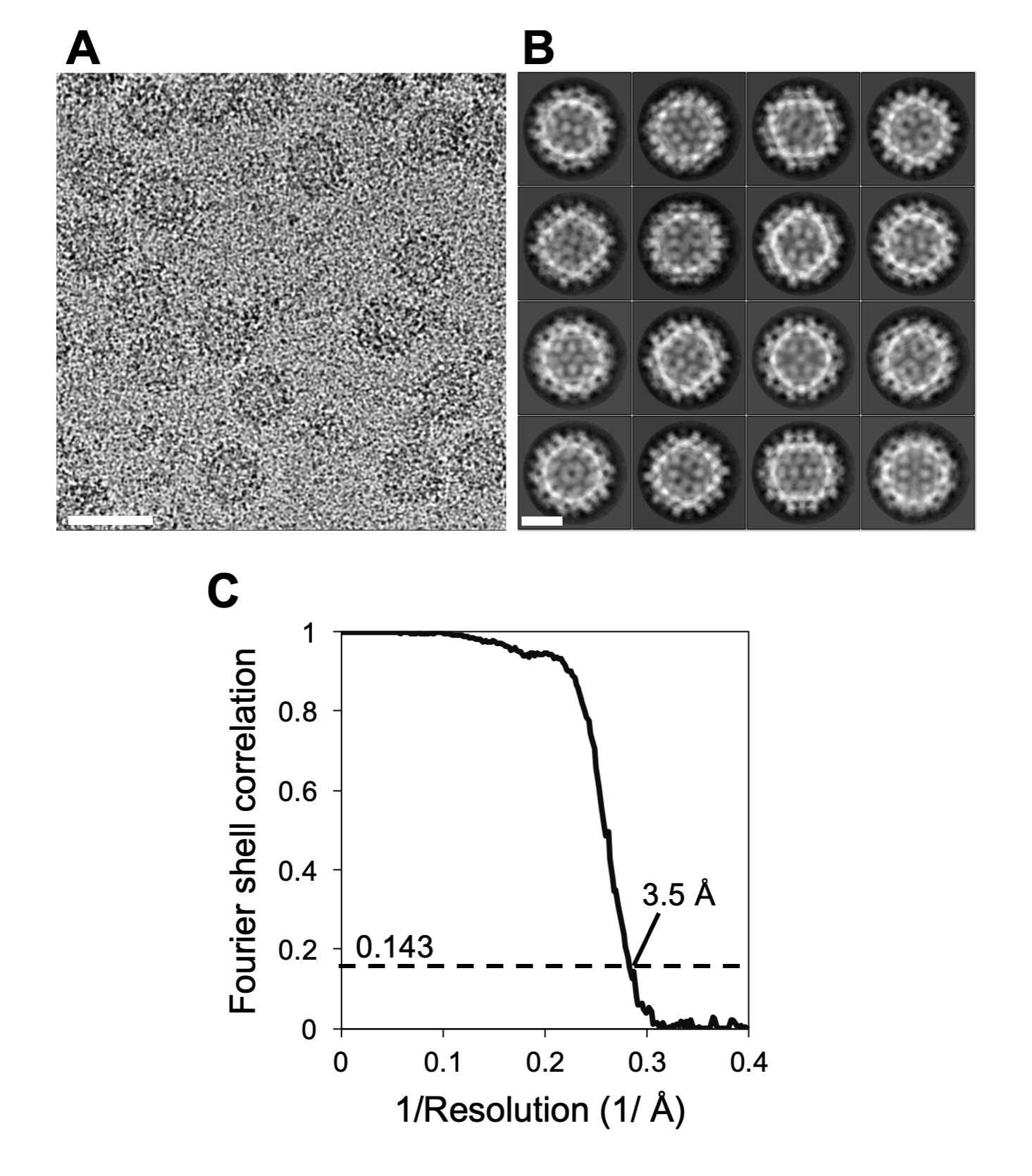

Supplement: S9 Fig — (A) Representative micrograph of MNoV-S7 VLPs. Scale bar, 500 Å. (B) An example of 2D class average images. Scale bar, 200 Å. (C) A GS-FSC plot. Based on the 0.143 criterion for comparing two independent data sets, the resolution of the reconstruction is estimated to be 3.5 Å. (TIF) [file ppat.1008619.s011.tif]

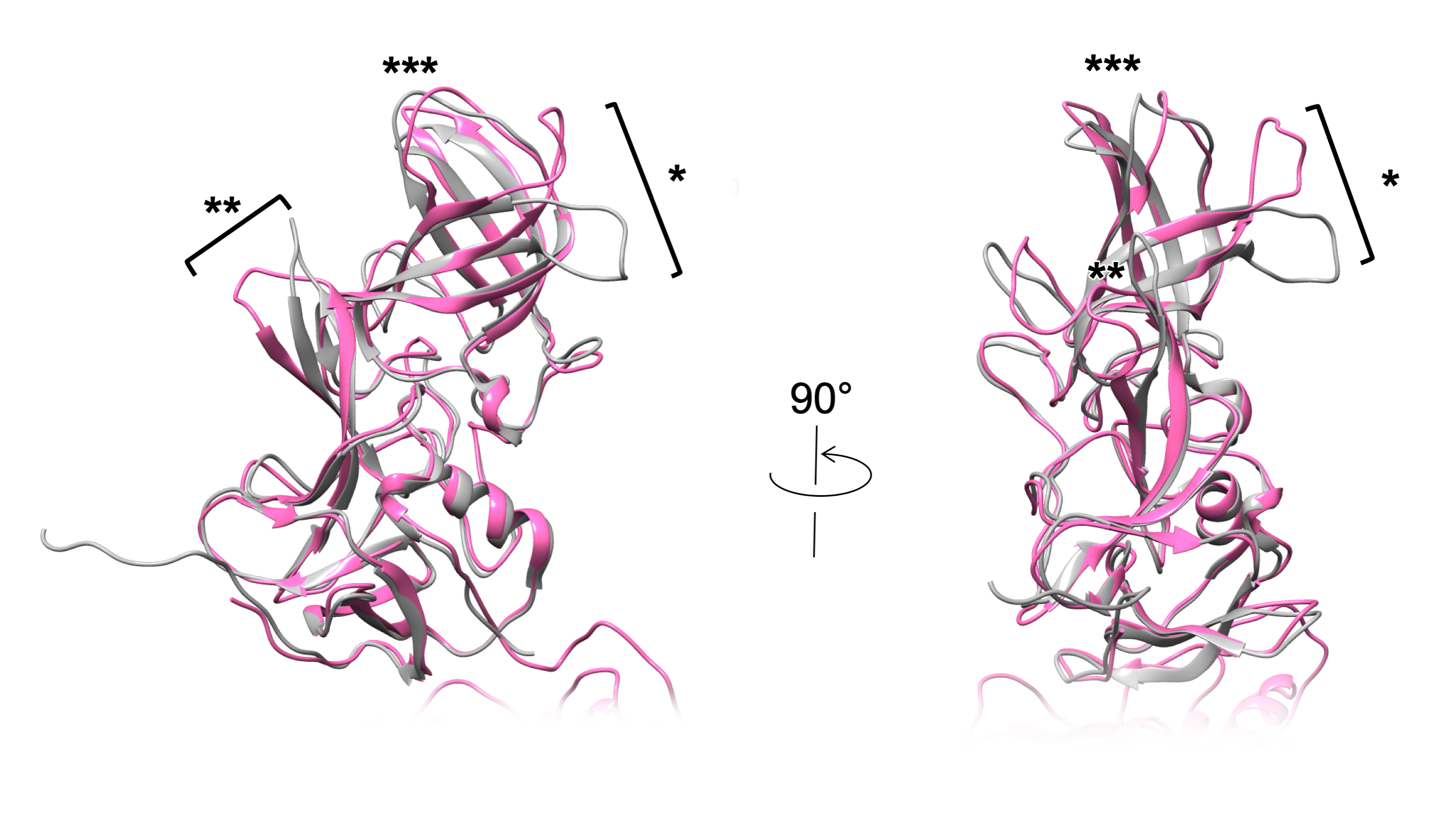

Supplement: S10 Fig — For the crystallographic model, 3QLE (PDB ID) was used, which is the model of the closed state in A–B loop and E–F loop. Ribbon diagrams of the cryo-EM map of the MNoV-S7 P domain and the crystallographic model of the MNoV-1 P domain are colored magenta and gray, respectively, which are shown in the different angles. The cryo-EM model of the MNoV-S7 P domain was analyzed in the aqueous condition of the resting P domain conformation, while the crystallographic model of the MNoV-1 P domain was expectedly analyzed in the aqueous condition of the rising P domain condition (in main text). The large structural difference was found in the βC''–βD'' loop (*) and the βC'–βD' loop (**) (S5 Fig), which make an interaction each other with those of the paired P domain in the P domain dimer. The cryo-EM model of the MNoV-S7 in DMEM shows the closed state in the βA”-βB” loop and βE''–βF'' loop (***) (S5 Fig). (TIF) [file ppat.1008619.s012.tif]

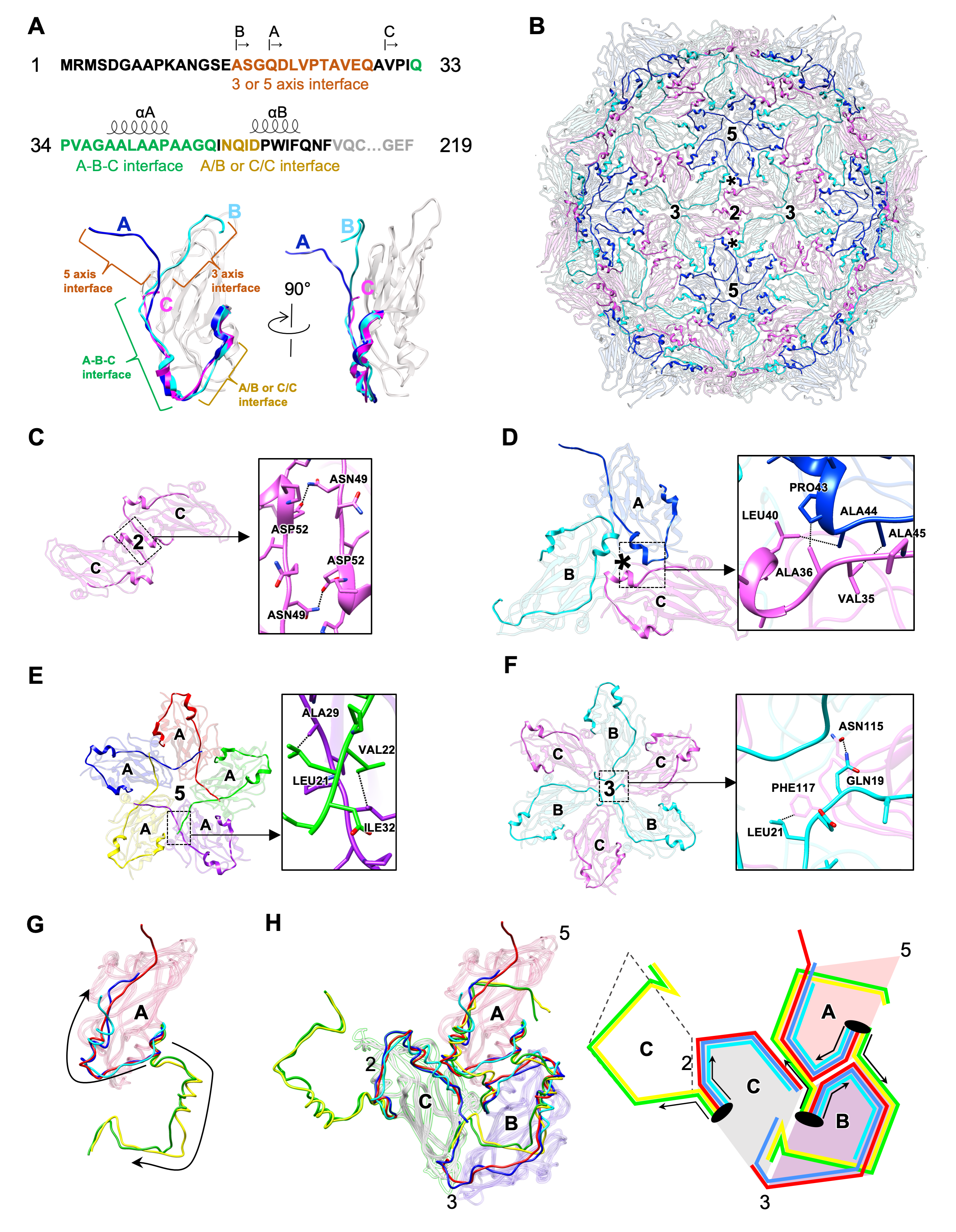

Supplement: S11 Fig — (A) Amino acid sequence corresponding to the N-terminal of the S-domain. The residues indicated by the arrows (Gln19, Ala16, and Val30) are the start residues of the sequence for modeling the respective VP1’s A, B and C monomers. Regions of the two short α-helices are indicated as αA and αB. In the lower panel, ribbon models of the S domain for the A, B and C monomers are superimposed. The residues colored blue, cyan, and purple correspond to the N-terminals of the A, B, and C monomers, respectively. (B) N-terminals of the A, B and C monomers on the S domain capsid are highlighted with the same colors in A. Asterisks indicate the pseudo-threefold axis of the T = 3 asymmetric units. (C) Interaction of the N-terminals of the S domains at the twofold axis. Asn49 and Asp52 of each C monomer form a polar interaction as shown by the dotted lines. (D and E) Interactions of the N-terminals at the pseudo-threefold axis in the T = 3 asymmetric unit and the fivefold axis, respectively. The counter residues at the adjacent N-terminals interact by hydrophobic bonds as indicated by the dotted lines. (F) Interaction of the N-terminal at the threefold axis. The N-terminal edge of the B monomer interacts with the adjacent loop in the C monomer by hydrophobic and polar interactions as indicated by the dotted lines. (G) Comparison of NTAs of the S-domain in the A monomer among the members of the Caliciviridae family: MNoV (red), HNoV GI.1 (blue, PDB ID: 1IHM), RHDV (cyan, PDB ID: 3J1P), SMSV (yellow, PDB ID: 2GH8) and FCV (Green, PDB ID: 3M8L). Arrows indicate the direction of the extended NTAs. (H) the adjacent B and C monomers were added in (G) to show the elaborate crosslinks between monomers. The right panel is a schematic figure of the left panel. The NTA of S domain in MNoV (red), HNoV (blue) and RHDV (cyan) runs along the bottom edge of its S domain and interacts with the adjacent S domains, while the NTA in SMSV (yellow) and FCV (green) runs along the bottom edge of the adjace [file ppat.1008619.s013.tif]

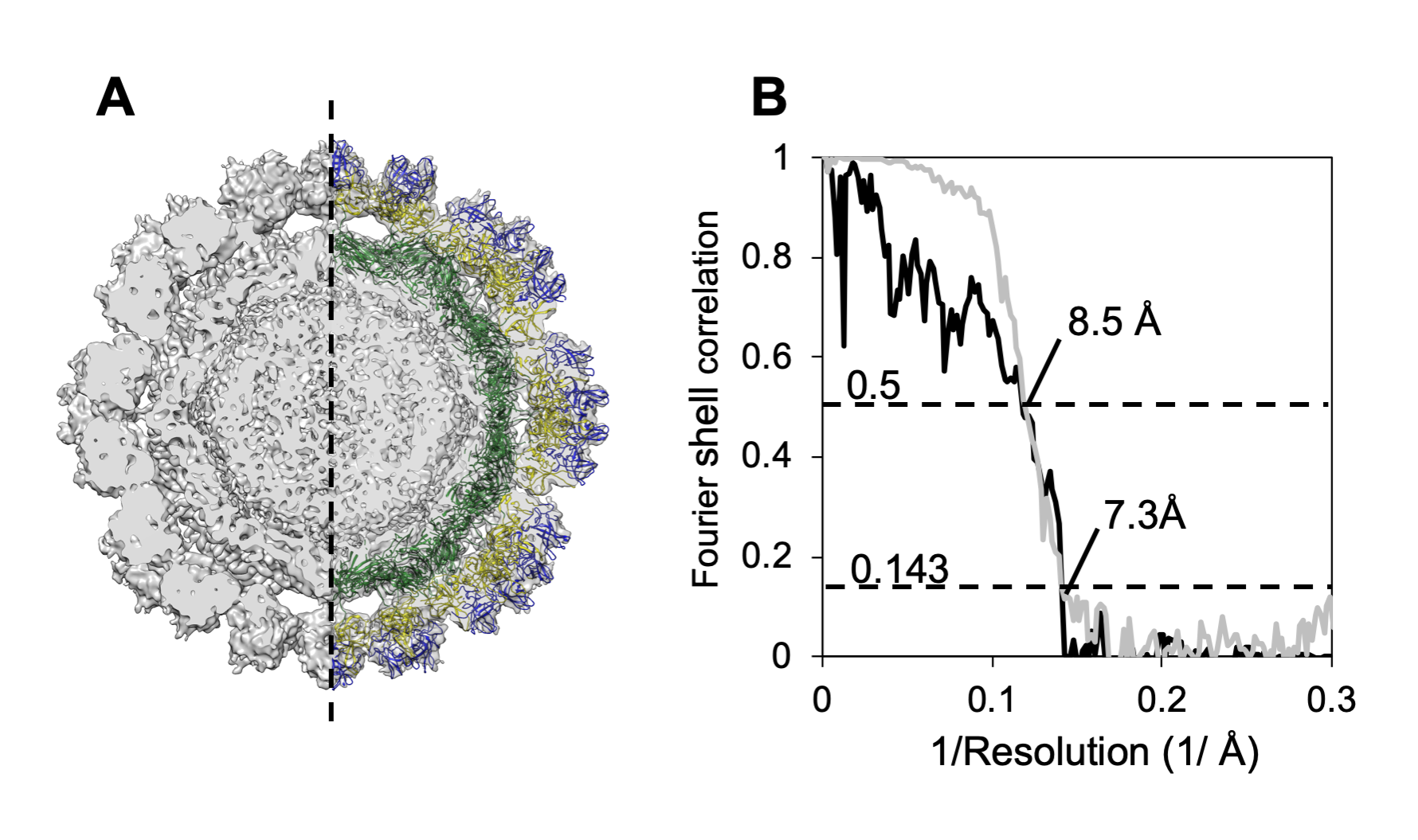

Supplement: S12 Fig — (A) The atomic model modified and fitted to the 7.2 Å cryo-EM map of the MNoV-S7 infectious particle suspended in PBS(-)+EDTA (pH 8) is shown on the twofold axis. The left side only shows the map. (B) The map to model FSC curve (black line) showing a similar curve with GS-FSC (gray) indicates 8.5 Å resolution at 0.5 criteria. (TIF) [file ppat.1008619.s014.tif]

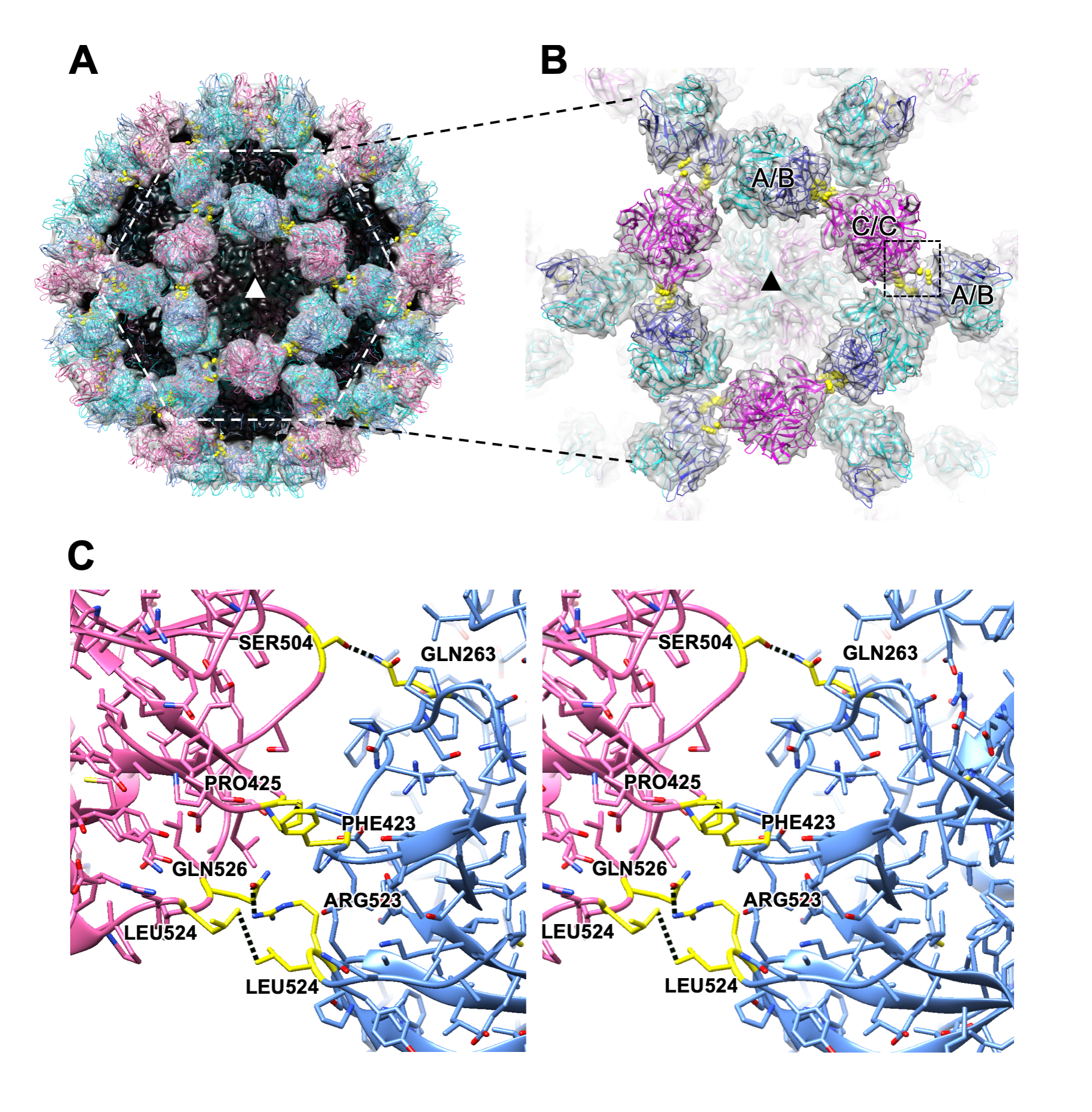

Supplement: S13 Fig — (A) A cryo-EM map of the MNoV-S7 with the rising P domain conformation is overlapped with the modified atomic model (S12 Fig). The black triangle shows the threefold axis. S domains are colored black. The residues forming interactions between the P domains are colored yellow. C/C-dimers (purple) on the twofold axis form interactions with adjacent A/B dimers (blue) located around the fivefold axis. (B) The enlarged view of the dotted hexagon in A. (C) A stereo view of the candidate chemical interactions between the C/C and A/B dimers indicated by the dotted square in B. Pro425, Ser504, Leu524, and Gln526 of the C/C dimer are facing with Phe423, Gln263, Leu524, and Arg523 of the A/B dimer, respectively (S5 Fig), where hydrophobic and polar interactions are suggested to be formed. (TIF) [file ppat.1008619.s015.tif]

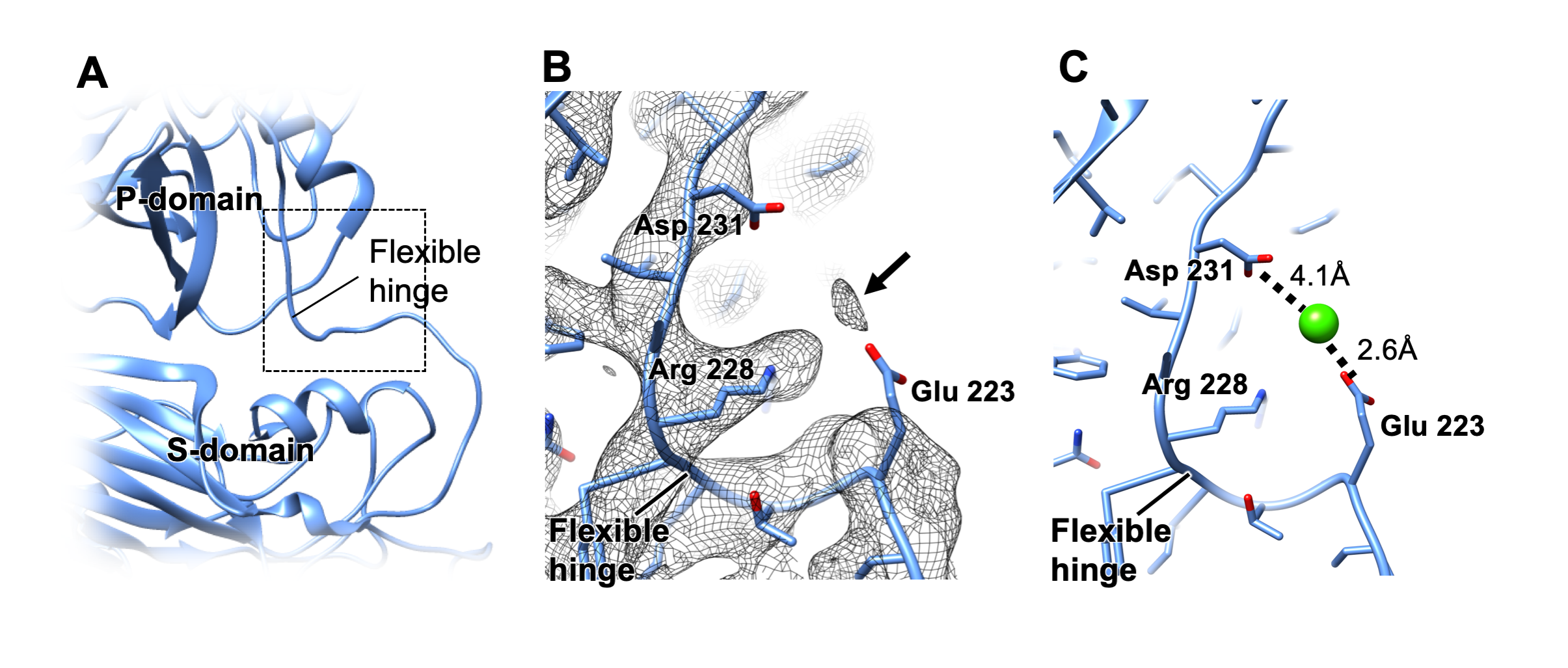

Supplement: S14 Fig — (A) An enlarged view of the flexible hinge connecting the S and P domains. The flexible hinge was indicated by the dotted box. (B) The enlarged map and model view of the box in A. Arrow indicates a density between Glu223 and Asp231. (C) Ca2+ as a possible metal ion is fitted into the density shown in B, and the distances from the near negatively charged residues are indicated. (TIF) [file ppat.1008619.s016.tif]

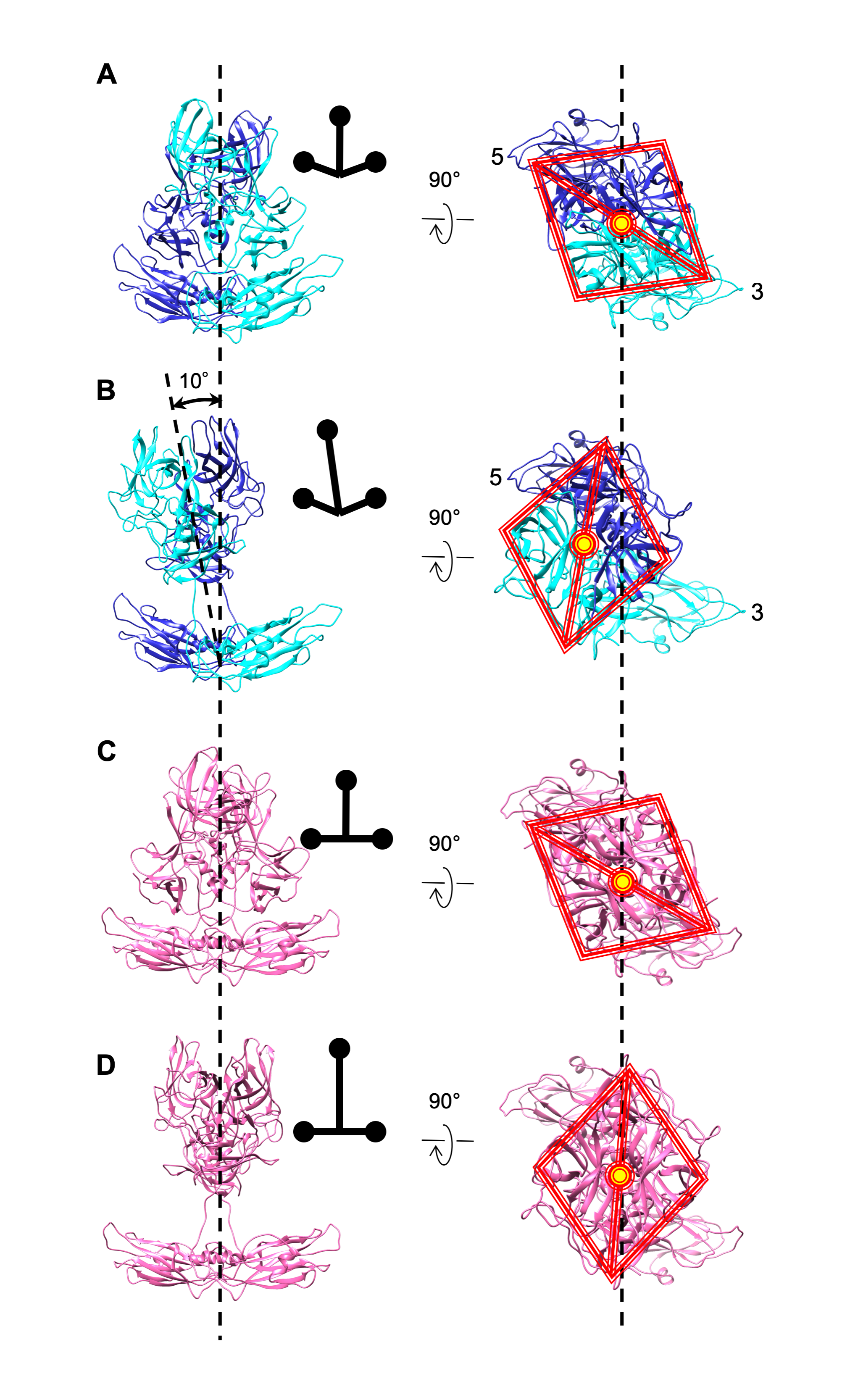

Supplement: S15 Fig — (A and B) The ribbon model of the A/B dimer in the resting and rising P domain conformations, respectively. The S domain dimers show a bent conformation. The P domain dimer is tilted 10 degrees toward fivefold axis in the rising conformation. (C and D) The ribbon model of the C/C dimer in the resting and rising P domain conformations, respectively. The S domain dimers show a flat conformation. The resting P domain dimer changes to the rising conformation without a tilt. (TIF) [file ppat.1008619.s017.tif]

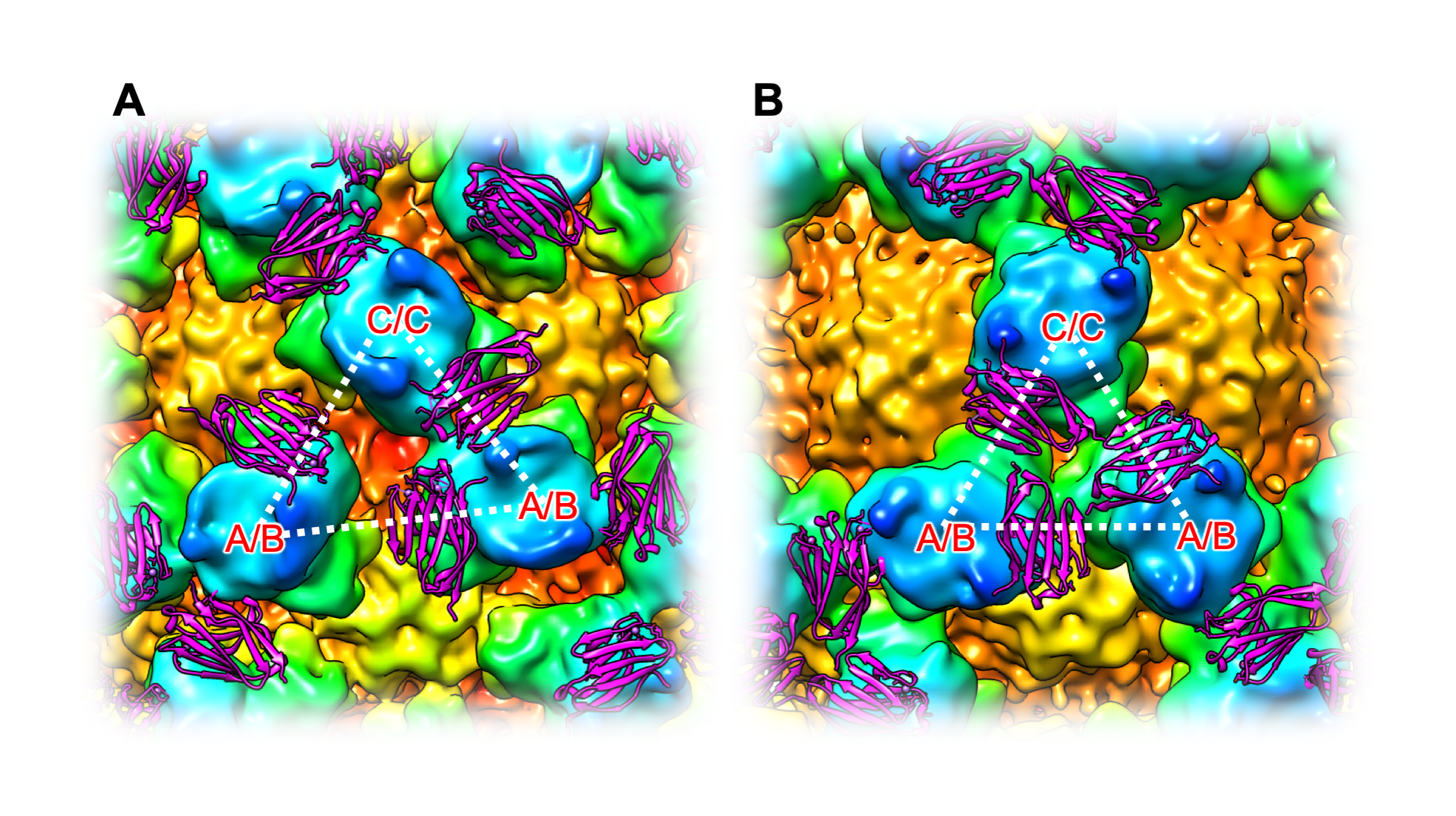

Supplement: S16 Fig — (A and B) The crystallographic model of the P domain and the CD300lf soluble domain (sCD300lf) (PDB ID: 5OR7) are fitted to the cryo-EM maps of MNoV-1 having the resting (A) and rising (B) P domain conformations, respectively. Three sCD300lf molecules (magenta) are able to bind on the P domain inside the triangle (white dot lines) formed between one C/C dimer and two A/B dimers. In the resting P domain conformation, the triangle is distorted and the CD300lf molecules are more flexibly able to approach the binding site (A), while in the rising P domain conformation, the triangle is squeezed, limiting the space for the sCD300lf molecules to approach the binding site (B). (TIF) [file ppat.1008619.s018.tif]

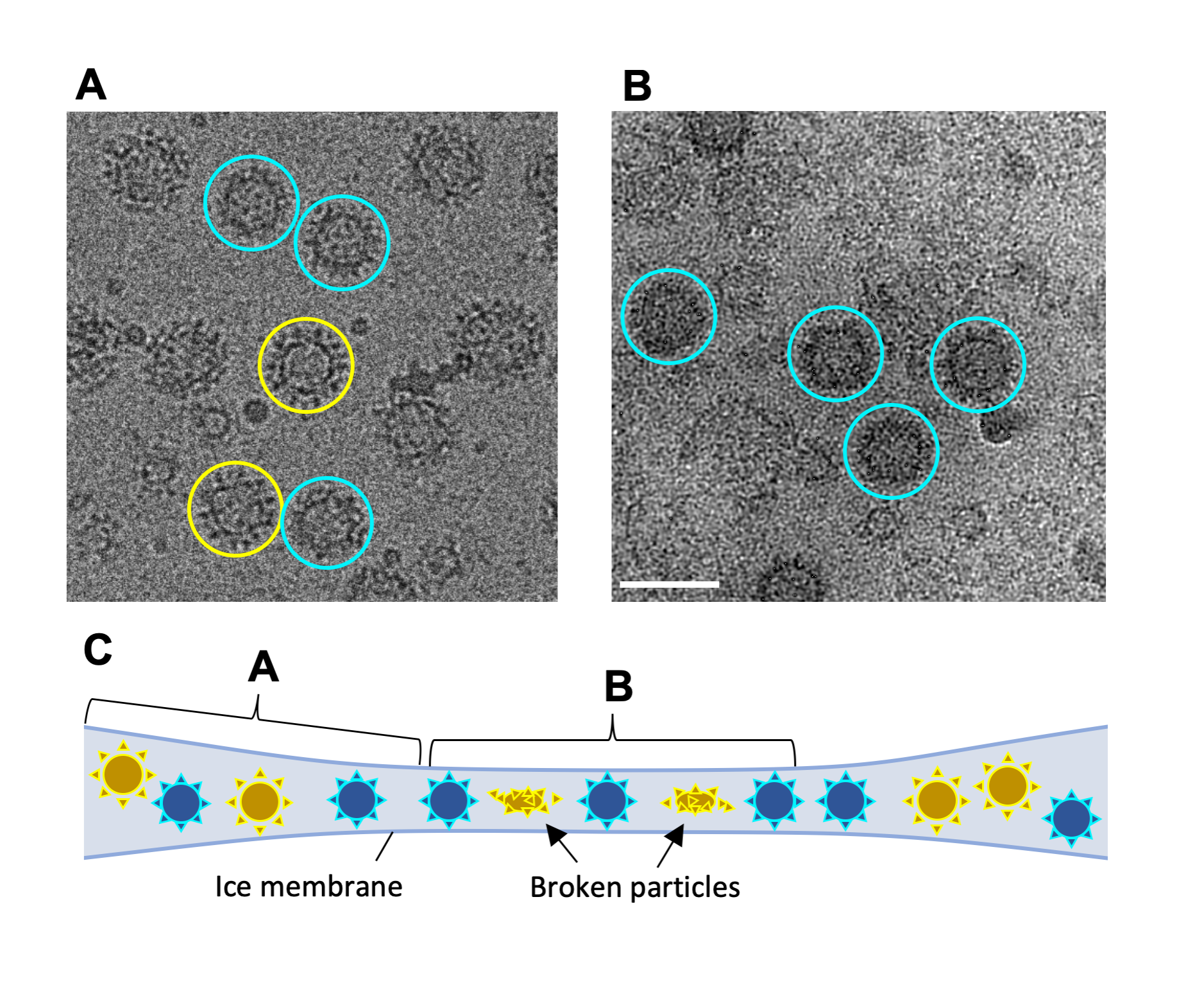

Supplement: S17 Fig — (A) Representative micrograph of the HNoV GII.3 VLPs having the resting (blue circles) or rising (yellow circles) P domain conformations. (B) The HNoV GII.3 VLPs having the rising P domain conformation easily collapsed due to surface tension in thin vitreous ice, and only the stable VLPs having the resting P domain conformations were left. (C) In the ice embedded cryo-EM grid, the ice film is formed in a convex shape, where the center is thinner than the hole edge. Eventually, the unstable particles move to the edge or collapse. (TIF) [file ppat.1008619.s019.tif]
