# Supplementary material for: Dynamic rotation of the protruding domain enhances the infectivity of norovirus
Source: PLoS Pathog. 2020 Jul 2;16(7):e1008619. doi: 10.1371/journal.ppat.1008619 (PMC7331980; doi:10.1371/journal.ppat.1008619)
Supplement: Attachment — Submitted filename: Response_to_reviewers_PPATHOGENS-D-20-00257-v13.docx [file ppat.1008619.s021.docx]

Author’s responses are colored in blue.

**Part II – Major Issues: Key Experiments Required for Acceptance**
Please use this section to detail the key new experiments or modifications of existing experiments that should be absolutely required to validate study conclusions.

Reviewer #1: Major:
- On July 04 2019 Snowden,..., Stonehouse published a study on biorxiv (https://www.biorxiv.org/content/10.1101/693143v1), 5 months before this study appeared on biorxiv. Here, also elevated P domains were observed in MNV, which rotated compared to the resting state. The study used heat treatment instead of pH. The results should be linked to and discussed with those presented here. In light of these results, l. 73/74 require rephrasing.

Authors’ response: Thank you for letting us know the latest reference. Snowden et al. only reported the resting state with several samples including heat treatment and compared them with previous rising conformation map (Katpally et al. 2010). Their results only showed the structural variability in the resting state. We have added the sentence below in Introduction (Line #80).

“During the preparation of this manuscript, Snowden and colleagues also reported the resting conformation of MNoV-1 infectious particles in several samples, including heat treatment, suggesting a potential for the conformational changes in a single virus [19], although the mechanism has not yet been elucidated.”

- Snowden et al reported a 3.1 A resolution structure. For the rising P domain, the P dimers had to be modelled separate from the shell. Little detail is provided by the authors, how they got to their 5+ A resolution structures. More detail on the reconstruction process has to be provided in the methods section. Moreover, was part of the dataset retained to check for overfitting of the data.

Authors’ response: First, the resolution reported by Snowden et al. needs to be questioned. In Fig. S2 of the manuscript, the local resolution of the shell extends to 1 Å. Given that in their data collection table (Table S1) they detail that micrographs were recorded at 1.065 Å, it is physically impossible to achieve 1 Å local resolution. The theoretical maximum would be 2.13 Å. Further, local resolution extends to 7 Å, but the P domains in Fig. S2 have no definition at all. For these reasons we are skeptical of their report.

We have added details regarding methods of 3D reconstruction and the model fitting into the rising conformation in S2 Table and S12 Fig. In S2 Table, we have included the numbers of initially picked particles in addition to the numbers contributed to the final map for each map. Further we have added the sentences including the percentages of the finally used particles in Discussion (Line #398 and #408). In S12 Fig, we have double checked a possibility of the overfitting of the model to the map by using map-to-model FSC, which was generated in the independent half map, in addition to the map and the fitted model.

- HNV is not generally used for human noroviruses, hNoV or huNoV should rather be used.

Authors: Thank you for your suggestion. We have changed HNV and MNV to HNoV and MNoV throughout text.

- In 2019, Mallagaray et al. reported a deamidation in some huNoV-like particles. This is strongly linked to pH. Have the authors any idea, whether MNV gets deamidated? Partial deamidation could alter kinetics and explain, why particles convert so slowly back to the resting conformation. Additionally, huNoV particles had been shown to often be instable at alkaline pH (e.g. Pogan et al. 2018). This was shown to be influenced by slight sequence variations as observed here for the two MNV strains (p. 10). This should be mentioned.

Authors: Thank you for your valuable information. At this moment, we have no data and references such deamination process occurred in the MNoV’s VP1. In our knowledge, the similar mechanism seems unlikely, because no compatible residues of asparagine 373 was not found in the sequences as shown in S5 Fig. On the other hands, pH sensitivity for the P domain’s rotation was slightly different between MNoV-1 and MNoV-S7 in which each induced the rotation at pH 7 and pH 8, respectively, though the difference of amino acids was only 18 residues. The situation is more likely to those of the HNoV VLPs as reported by Pogan et al. 2018. We have added the sentences below in the text (#204 and Line #357) and included the references.

“HNoV particles have often been shown to be unstable at alkaline pH, while sensitivity has been shown to vary significantly between genotypes and to be affected by slight sequence variations [25]. Differences in pH sensitivity between the two MNoV strains may also follow a similar mechanism.”

“In HNoV GII.4, post-translational modification of Asp373 by a deamidation was reported to attenuate histo-blood group antigen (HBGA) binding with an estimated half-life of a few days [35]. The slow reaction is also strongly linked to pH. However, it seems to be a different mechanism from the P-domain rotation in MNoV, as no compatible residues of Asp 373 were found in the sequences (S5 Fig), in addition to the fact that glycan binding is not essential for infection of MNoV.”

- Jung et al. 2019 also observed rising and resting conformations in huNoV-like particles. Even more relevant to the here presented study Koromyslova et al. observed in 2015 structural alterations in presence of citrate (Treatment of norovirus particles with citrate), which resemble a lot the micrographs of the rising domain in Fig. S1. Do the authors consider these structures similar? It would present alternative strategies to achieve the rising conformation in huNoVs, which are not known to bind cations. What is the fraction of broken/disassembled particles observed?

Authors: Thank you for your suggestion. We believe that the rising and resting P domain conformations shown in HNoV VLPs (Jung et al 2019) represent one conformation of the two as we showed in HNoV GII.3 VLP in our study. However, we have not succeeded to induce the alternative conformations in HNoV at this moment. We guess it has more complicated mechanisms compared to MNoV. On the other hand, we think it works with the different mechanism in the expanded VLP in citric acid reported by Koromyslova et al 2015, because we have confirmed the GII.10 VLP originally represented the rising conformation in the aqueous condition (Hansman et al., J Viol, 86:3635 2012). In our experiments, we observed some broken/disassembled particles. Especially, the damaged particles were more identified in the rising conformation as we describe it in Line #398. Therefore, we concluded that the particles having the rising conformation is more flexible than the resting conformation ones. To explain the facts mentioned above, we have added sentences in Discussion (Line #348) with the references.

“So far, the resting and rising conformations were independently observed in several HNoV VLPs [32]. However, this report is the first case to present two conformations simultaneously in the same particle in a reversible state. Koromyslova et al. reported the structural alterations in presence of citrate in HNoV GII.10 VLP [34], which strongly resemble the micrographs of the rising P domain in S1E and S6G Figs. We, however, believe the mechanism works differently mechanism in the expanded VLP in citrate, because we have confirmed the GII.10 VLP originally represented the rising conformation in the aqueous condition and the resting conformation has not yet been identified [13].”

- The authors only touch briefly on VP2 and genome. It should be stated more clearly if in any of the conformations of MNV-1 or MNV-S7 virions extra density was observed in asymmetrical reconstructions that could be attributed to VP2 or genome-protein contacts.

Authors: Unfortunately, we have not found the structure of VP2 in our icosahedral-symmetrized cryo-EM map in this study. When a similar mechanism of feline calicivirus (Conley et al. 2019) is applied to MNoV, twelve copies of VP2 dispersed within particles could accumulate and be assembled in response to changes in MNoV structure. We have added sentences below in Discussion (Line #423).

“As with FCV, no VP2 density was found in our symmetry-imposed cryo-EM map of noroviruses. One reason is that the copy number is limited and does not match the icosahedral symmetry, where the components disappear due to imposition of symmetry. Assuming the same mechanism as FCV, VP2s in noroviruses dispersed inside the particle could accumulate and be assembled according to the structural changes due to receptor involvement.”

- DMEM is rather complex compared to PBS. It would be important to also show particles in PBS +/- EDTA at pH 8 and also at lower pH to more clearly pinpoint the effect to pH.

Authors’ response: Thank you for your suggestion. We have added the data of PBS(-) +/- EDTA in Fig. 1E and S6K Fig, in which PBS(-) without EDTA was the similar effect with DMEM. In addition, we have also added sentences below in Results (Lines #116 and #192).

“where the rotation from the rising to resting conformation was simply triggered at a pH lower than 7 (Fig. 1E).”

“The reversible rotation from the rising to resting conformation was also triggered at a pH lower than 8 (S6K Fig).”

- The structural conversion is super slow with 2-8 h and also the mixed species that are observed are super-interesting. Have the authors a theory for the slow kinetics? Could the P domain connections stabilize a given conformation causing hysteresis? While these aspects are discussed, they warrant a more thorough reflection in terms of potential mechanisms, timeline of infection (binding, entry and genome release). I hence recommend extending the discussion of these aspects.

Authors’ response: Thank you for your suggestion. We agree with the idea “the P domain connections stabilize a given conformation causing hysteresis”. By the way, the kinetics depends on the aqueous condition, especially pH gradient. In the condition of ~pH 7, it takes 2-8 h. However, in lower pH condition, the reaction is accelerated (less than 5 minutes) in our observation. It may reflect the dramatical environmental changes in the gastrointestinal tract. We believe that the slow kinetics in MNoV is somewhat related to the 4th major comment from the same Reviewer, in which the slow reaction of a deamidation in HNoV P domain control the infectivity and strongly linked to pH. We have modified the sentence and added more explanations as below in Lines #166 and #357.

“The mixed P domain structure within a single particle suggests that even if the conversion of individual P domains may be rapid, it would take more time to modify the overall conformation of the capsid, where the P domains interact and are connected together like an entangled net, in which the P domain connections stabilize a given conformation causing hysteresis. In our observation, the transformations between the resting and rising P domain conformations were accelerated in higher or lower pH conditions, where they were completed in less than 5 minutes at the fastest, respectively.”

“In HNoV GII.4, post-translational modification of Asp373 by a deamidation was reported to attenuate histo-blood group antigen (HBGA) binding with an estimated half-life of a few days [35]. The slow reaction is also strongly linked to pH. However, it seems to be a different mechanism from the P-domain rotation in MNoV, as no compatible residues of Asp 373 were found in the sequences (S5 Fig), in addition to the fact that glycan binding is not essential for infection of MNoV. The slow regulation step to control infectivity may be common between MNoV and HNoV and essential for infection in the dramatic environmental changes present in the gastrointestinal tract, though the approaches are different.”

- What was the reasoning to use such closely related MNV strains? A more distant MNV would have been of higher interest to establish a general relationship between rising/resting conformation and infection.

Authors’ response: It is a historical reason. We originally studied the structural difference between infectious particle and VLP in MNoV-S7, because we have both infectious particles and VLPs. However, we accidentally found the conformational difference of the P domain with the previously reported MNoV-1 cryo-EM structure (Katpally et al. 2010). We started to analyze the structure of MNoV-1 with our sample condition. While the MNoV-1 VLP exhibits two types of VLP (T=4 ~48nm particle and T=3 ~40 nm particle) as shown in S8 Fig, the MNoV-S7 VLP shows a single T=3 ~40 nm particle. Hence, we used MNoV-S7 VLPs for high-resolution structural analysis in addition to the biosafety reason. These have already been written in the main text. On the other hand, we used MNoV-1 particles for comparison with previous report. However, we were fortunately able to find the difference of pH sensitivity for dynamic rotation of P domain between MNoV-1 and MNoV-S7, though these strains are closely related. We have added more explanations below in Results (Line #186).

“Originally, we used this strain for structural study because we were already able to produce both the infectious particle and VLP [20]. However, since the P domain structure of the particle acquired was different from the previously reported cryo-EM map of MNoV-1 [14], MNoV-1 was used to confirm the structure in our sample preparation method.”

- 40-50 nm VLP of MNV-1 suggests assembly defects or formation of T=4 particles. Those have been observed in huNoVs previously, e.g. in GII.4 strains by Devant et al 2019. While the larger particles can be the result of suboptimal production conditions, it would be of interest to show those micrographs and explain what the larger particles are.

Author’s response: Thank you for your suggestion. We have added the structural of 40-50 nm VLPs of MNoV-1 as S8 Fig.

- Upon lifting and rotation, are there any structural changes within the P domain backbone? This should be mentioned explicitly.

Authors’ response: We have not had an high-resolution structure of the rising P domain conformation. Hence, we cannot discuss the structural difference at atomic level. We fitted the atomic model of resting P domain to the rising one and calculated the map to model FSC to evaluate the over fitting. It showed the similar curve with GS-FSC representing 8.5 A resolution with 0.5 criteria. This suggests that the atomic model of the resting P domain is very similar to the rising P domain. However, when we compared our cryo-EM model of the MNoV-S7 VP1 in the resting P domain conformation with the crystallographic model of MNoV-1 P domain (3QLE and 5OR7) expected to be folded in the rising P domain aqueous condition, we found some structural differences in the connecting loops in the P domain dimers. We have added the sentences below in Results (Line #234 and #290) and added the “map to model FSC” in S12B Fig.

“”

“A comparison of the atomic model of the MNoV-S7 VLP with the reported crystallographic models of the MNoV-1 P domain (PDB ID: 3LQ6 and 3LQE), which were expectedly analyzed in the aqueous condition of the rising P domain conformation [29], shows several interesting results. First, our cryo-EM model of the P domain in MNoV-S7, which was analyzed in the aqueous condition of the resting P domain conformation, had a higher degree of structural similarity to 3LQE than 3LQ6 (S10 Fig), suggesting that the cryo-EM model dominantly exhibits the closed state between the βA''–βB'' loop and the βE''–βF'' loop (S5 Fig, triple asterisks in Fig 3E and S10 Fig). Second, the βC''–βD'' loop (S5 Fig) extends upward with respect to the capsid surface (single asterisk in Fig 3E and single asterisk of magenta residues in S10 Fig) and forms an interaction with the βC'–βD' loop (S5 Fig, double asterisks in Fig. 3E, and double asterisks of magenta residues in S10 Fig) of the paired P domain in the P domain dimer in our cryo-EM model, whereas the βC''–βD'' loop (S5 Fig) in crystallographic models of MNoV-1 (3LQE than 3LQ6) extends downward with respect to the capsid surface (single asterisk of gray residues in S10 Fig) and form an interaction with the βC'–βD' loop (S5 Fig, double asterisks of gray residues in S10 Fig) of the paired P domain in the P domain dimer. Intriguingly, these two loops were disordered in the recent reported crystallographic model of the MNoV-1 P domain dimer containing the soluble domain of the cellular receptor, CD300lf (PDB ID: 5OR7) [30]. The facts suggest that an allosteric effect in the P domain [31] may exist during the dynamic P domain rotation and the receptor binding, though we have not had the detail structure of P domain in these conformations yet.”

“The atomic model of the MNoV-S7 VLP in DMEM was modified and fitted into the 7.2 Å cryo-EM map of the MNoV-S7 infectious particle suspended in PBS(-)+EDTA (pH 8) with the high cross-correlation coefficient of 0.92 (S6I- S6J and S12A Figs) (see Materials and Methods). The map to model Fourie Shell Correlation (FSC) showing a similar curve with “gold standard”-FSC (GS-FSC) indicated 8.5 Å resolution at 0.5 criteria (S12B Fig). This suggests that the atomic model of the resting P domain conformation is very similar to the rising P domain conformation at the resolution.”

- Have the authors an idea why CD300lf binds so poorly? Have they tested whether bile acids or glycans enhance the binding of the protein receptor? This could be discussed in a bit more detail. How would the reconstruction look without imposing symmetry? Maybe the receptor would be less blurred out?

Authors’ response: As you mention, our cryo-EM map of MNoV with CD300lf was unfortunately poor. It may be necessary to add some co-factors like bile acids and glycans as you suggest. According to the Reviewer #2’s comment, we have removed the result of CD300lf, and continued the discussion using the previously reported X-ray crystallographic models of MNoV-1 and sCD300lf (PDB ID: 5OR7). We have replaced the sentences (from Line #366) and S16 Fig.

- Fig. S9C: electron density seems to indicate a subunit interaction that is not well reflected in the model, i.e. Ser-Gln maybe positioned lower or are in fact other residues interacting? The two lower interactions lack density entirely. How certain are these?

Authors’ response: Thank you for letting us know the important point. In the figure, the atomic model of the resting P domain conformation at 3.5 Å resolution was rigid-body fitted into the 7.2 Å resolution cryo-EM map of the rising P domain conformation, where the small side chain densities are missing in the low-resolution map. Thus, the interaction is a speculation based on the fitted model. We have changed the sentence as below in Results (Line #299) and removed the cryo-EM map from the figure (S13 Fig).

“The atomic model fitted to the cryo-EM map of the rising conformation suggested that a new interaction links the P1 subdomain of the C/C dimer to the adjacent P1 subdomain of the A/B dimers via hydrophobic and polar interactions (S13C Fig).”

- How different are the positions of the rising P domain from each other in the three different strains/particles? An overlay would be useful.

Authors’ response: The cryo-EM maps of the virus particles having the resting (~5 Å resolution) and rising (~7 Å resolution) P domain conformations are very similar between MNoV-1 and MNoV-S7 (cross-correlation coefficient > 0.91). There is no significant structural difference at each resolution level. Therefore, we have just compared MNoV-1 and HNoV GII.3 in S4 Fig. In the two structures, the angle of rotation between the resting and rising P domains is coincident (~70° clockwise), and the interactions between P domain dimers are similarly observed at the P2 levels in the resting conformation and at the P1 levels in the rising conformation, respectively. However, the heights of the P domain are significantly different in the rising P domain conformations, where the P domain of HNoV GII.3 is ~4 Å lower than MNoV-1. We have added S4 Fig and added the sentences below in Results (Line #152).

“The structure of the resting and rising P domain conformation was compared in more detail between MNoV-1 and HNoV GII.3 (S4 Fig). In the two structures, the angle of rotation between the resting and rising P domains was coincident (~70° clockwise), and the interactions between P domain dimers were similarly observed at the P2 levels in the resting conformation and at the P1 levels in the rising conformation, respectively. However, the heights of the P domain are significantly different in the rising P domain conformations, where the P domain of HNoV GII.3 is ~4 Å lower than MNoV-1. The length of the hinge loop between the S and P domains are the same in these NoVs (S5 Fig). It suggests that the holding manner in the hinge loop is different between MNoV and HNoV.”

As side note, with the wealth of supplementary data and the caption being given after the references, it was difficult to review the manuscript. I would have appreciated two files and captions closer to the figures.

Authors’ response: We are sorry for the inconvenience. It has been fixed in the revision.

Reviewer #2: (No Response)

Reviewer #3: None

**Part III – Minor Issues: Editorial and Data Presentation Modifications**
Please use this section for editorial suggestions as well as relatively minor modifications of existing data that would enhance clarity.

Reviewer #1: Minor:
- last sentence of abstract is difficult to understand and requires rephrasing

Authors’ response: We have tried to rephrase the last sentence of Abstract explicitly, as follows (Line #30).

“These results provide new insights into the mechanisms of non-enveloped norovirus transmission that invades host cells, replicates, and sometimes escapes the hosts immune system, through dramatic environmental changes in the gastrointestinal tract.”

- l. 221 vs. l. 227 appear contradictory. The fine structure discussion is not fully clear from the phrasing.

Authors’ response: Thank you for letting us know the inconsistency. NTA of MNoV is not completely unique, which belongs to one of two groups we classified. However, we found a new feature of NTA in A monomer. We have modified the sentences as below from Line #277 to make them clear.

“As shown in S11G-S11H Figs, the previously reported NTAs of caliciviruses are structurally divided into two groups. One group, belonging to norovirus and lagovirus shown in HNoV GI.1 [7] and RHDV [15], is that the NTA runs along the bottom edge of its S domain and interacts with the adjacent S domains (blue and cyan in S11G-S11H Figs). The other group, belonging to vesivirus shown in SMSV [16] and FCV [17] is that the NTA runs along the bottom edge of the adjacent S domain and interacts with the adjacent S domains (yellow and green in S11G-S11H Figs). NTA in MNoV has a similar conformation as the first group (red in S11G-S11H Figs), further clarifying the function of the NTA in the A monomer. Consequently, the rigid capsid shell of MNoV was maintained by these outer P domain interactions and inner S domain crosslinks.”

- l. 500 A/B dimer not diner

Authors’ response: We have corrected the typo.

- Fig. S1 caption says PBS(-)-EDTA whereas figure says +EDTA. The latter makes more sense to the referee. The authors should check whether this has been correctly stated throughout text.

Authors’ response: Thank you for pointing out the contradiction. We have fixed all phrases to PBS(-)+EDTA in text and figures.

- Fig. S6: comparing MNV sequences to huNoV GII.3 would be more useful to estimate how different these are. What is the rationale for showing the Norwalk sequence?

Authors’ response: Thank you for your suggestion. We initially present the Norwalk sequence because we have used it for homology model building of the S domain. We have further added the sequence of HNoV GII.3 for sequence comparison in new S5 Fig.

- Fig. S10: please also label the other residue present in B and C. Is it an Arg and how far is its functional group away from the putative Ca2+? There is an additional residue, presumably from the helix, overlaying with Asp 231. If this is not close to the metal binding, consider taking this out to increase clarity of the view.

Authors’ response: Thank you for your suggestion. We have fixed the new S14 Fig, in which all polar residues near the possible metal density are labeled, and the only negatively charged residues of Asp and Glu include their distances to the possible metal density.

Reviewer #2:

1) I suggest merging figures 1 and 5 so the reader grasps at once that the two conformations are found in both MNV-1 (for which only Ri was reported so far) and GII.3 VLPs.

Authors’ response: Thank you for your suggestion. We have merged the Fig. 1 and 5 and made a new Fig. 1. According to this, the order of the paragraphs has also changed in Results.

2) Count of particles in Ri and Re conformations: This is another important piece of evidence. Both conformations are found on the same grids and depending on buffer conditions their relative abundance changes (appendix figures S1 I, S4 K). Furthermore a time course of going from Ri to Re is actually given for MNV-1, with indication that mixed particles (part Ri, part Re) can be detected (S3 Fig).

a) Most important: How are the particles counted ? I could not find that information in the paper. I am guessing from e.g. S3 Fig. that during image processing 2D classes are used to assign particles to Ri, Re or mixed. Or was this done by eye on random subsets of extracted particles ? If so, which set of particles was used to draw the subsets ? Please give a clear and detailed description of the process.

Thank you for your suggestion. Because the 2D class average images of the particles having the resting and rising P-domain conformations are distinctly different as shown in S1B, S1F, S6D, and S6H figs, the number of particles was counted in the classified 2D images. We have included the sentence as follows in the legends of Fig 1E and S6K Fig. “The number of particles in each conformation was counted during image processing 2D classes.”. In the case of old S3 Appendix A (currently Fig 2A), after 2D classification, only the particles in the classes showing the resting conformation in all P-domains were counted in each time point. We have included the sentence as follows in the legend of Fig 2A. “The particles showing the resting conformation in all P-domains were counted in the classes after 2D classification.”

b) On a related note, very few particles per image are actually used in the 3D reconstructions. For instance l. 448 'For MNV-1 infectious particles suspended in DMEM and PBS(-)-EDTA (pH 8.0), 6,708 and 4,704 particles were selected from 1,046 and 2,188 images, respectively'. Yet e.g. the inserts from micrographs in S1 Fig. show 6-12 seemingly good particles, so most are rejected during image processing. Please give numbers: How many 'not junk' particles (seemingly good particles) don't make it to the final 3D reconstruction ? Discuss in terms of heterogeneity (mixed spike conformations, departure from icosahedral symmetry, broken particles, ...).

Authors’ response: Thank you for your suggestion. We have added the number of the initially selected particles in S1 and S2 Tables in addition to the number of the particles contributed to the final map. As suggested, mixed spike conformations, departure from icosahedral symmetry, and broken particles were excluded during 3D map refinement because they reduce the final resolution. We further have added sentences including the percentages of the finally used particles as below in Discussion (Line #398 and #408).

“In addition, the particles finally contributed to the 3D map were 23~28% of the initially picked particles in the rising conformation data set, while they were 91~95% in the resting conformation data set (S2 Table), where mixed P domain conformations, departure from icosahedral symmetry, and broken particles were removed during iterative 2D and 3D map refinements.”

“In the case of HNoV GII.3 VLPs, the particles finally contributed to the 3D map was 29% of the initially collected particles in the rising conformation data set, while they were 50% in the resting conformation data set (S2 Table).”

c) Make a separate, main figure from appendix figures S1 I, S4 K and S3. This could be new Fig. 2.

Authors’ response: Thank you for your suggestion. We have made new Fig. 2, which include old S3 Fig. Old S1I and S4K Figs were inserted in Fig. 1 and new S6 Fig, respectively, because these data indicate the aqueous condition of dynamic P domain rotation in MNoV-1 and MNoV-S7.

3) l. 345 'HNV GII.3 VLPs also showed that the rising P domain conformation easily collapsed due to surface tension of thin vitreous ice, thus leaving most of the particles with a resting P domain conformation in the thin ice of cryo-EM (S14 Appendix).'
l. 896 'The VLPs having the rising P domain conformation easily collapsed due to the surface tension in thin vitreous ice, and only the stable VLPs with the resting P domain conformations were left.'
I do not understand the basis of these statements. Do the authors suggest that the spikes collapsed between sample deposition and plunge freezing ? Why ?

Authors’ response: In thinner ice film, the particles are subjected to strong mechanical stress due to surface tension. In the ice embedded grid used in cryo-EM, the ice film is formed in a convex shape, where the center is thinner than the hole edge. Eventually, the unstable particles move to the edge or collapse. We have added the schematic figure and the description as S17C Fig.

4) l. 291-314 in the discussion section actually belong in the results.

Authors’ response: Thank you for your suggestion. According to your suggestion in the comment 6), we have removed the discussion and added a new discussion using the previous reported crystallographic model.

5) l. 276 'metal ions binding to the region between the P and S domains, may help to stabilize the resting conformation (S10 Appendix).'

The evidence for this is that EDTA-containing buffers seem to favor Ri, while DMEM favors Re. However, pH seems to be the most important factor.

a) What is the metal ion content of DMEM ?

Authors’ response: DMEM includes the inorganic salts of CaCl2, (Fe(NO3)3"9H2O, MgSO4, KCl, NaHCO3, NaCl, NaH2PO4-H2O. We have modified the sentence as below (Line #338).

“These residues may function to stabilize the resting conformation via metal ions in DMEM, which includes many inorganic salts such as CaCl_2_, (Fe(NO_3_)_3_･9H_2_O, MgSO_4_, KCl, NaHCO_3_, NaCl, NaH_2_PO_4_-H_2_O [33].”

b) It could be worth mentioning that the GI.1 crystal structure, until very recently the only Re conformation, was obtained at pH4.8.

Authors’ response: Thank you for your suggestion. We have added the sentence below in Discussion (Line #320).

“So far, only one of two conformations has been reported in each HNoV VLP (resting conformation: GI.1 (pH4.8), GI.7, GII.2; rising conformation: GII.4, GII.10) and other caliciviruses [11,13,32].”

c) l. 279 'we identified density between the two carboxylates (Glu223 and Asp231) in the flexible hinge of the resting P domain conformation, where a metal ion is expected to bind to these residues (S10 Appendix)'. The density shown in S10 Fig is not convincing. At this threshold the map shows other unexplained features, suggesting that it is contoured too low. Please remove this statement.

Authors’ response: As pointed out, this data needs higher resolution to clearly identify a metal density. We have thus removed this statement, and added the new sentence below in a lower tone (Line #331).

“As a candidate, we found a potential interaction via a metal ion between two carboxylates (Glu223 and Asp231) observed in the folded, flexible hinge of the resting conformation (S14 Fig).”

6) The CD300lf data are not convincing.

a) l. 875 'rCD300lf is fitted to the extra density using the docking simulation with a parameter set shown in Materials and Methods'. If I understand correctly, the authors perform ab initio docking, find a pose that roughly matches the extra density, and conclude that they have the approximate position of the receptor binding site. However, the extra density is clearly not that of rCD300lf and the docking pose does not match the crystal structure of the complex of the receptor with the spike (Kilic et al., J. Virol. 2018). This part should be discarded entirely.

Authors’ response: Thank you for your suggestion. We have discarded this part entirely.

b) Discussion of the accessibility to rCD300lf in Ri vs Re is relevant here but should simply match the available crystal structure to the Re and Ri reconstructions.

Authors’ response: Thank you for your suggestion. We have changed the discussion using the previously reported crystallographic model (from Line #366).

c) l. 329 'The resting P domain conformation showed higher accessibility of the cellular receptor CD300lf than the rising P domain conformation, causing greater viral infectivity.' As I remember Kilic et al. did not find any hindrance to binding to the MNV (Ri) conformation. But even then the 'causing greater viral infectivity' would be speculative.

Authors’ response: Thank you for your suggestion. Kilic et al. (2018) reported “the sCD300lf slightly clashed at the P dimer interactions, suggesting that multiple sCD300lf molecules might not bind simultaneously at this interaction”, when they superimposed sCD300lf into the cryo-EM structure of the MNoV-1 virion based on their crystal model. Nelson et al. (2018) also wrote “This docking model results in some minor overlap of CD300 domains (Fig. 7C), which may be resolved by mobility of P domains, which are flexibly connected to the shell domain of VP1.” Furthermore, Snowden et al. (2019) recently described “Interestingly, a clash between trimers of CD300lf in the previous docking model (Nelson et al. 2018) is resolved in this model, and there is space for higher receptor occupancy.” when they fitted the crystal model into their resting state cryo-EM map of MNoV-1. In this revision, we used a crystallographic model (PDB ID: 5OR7) to confirm the difference in the physical affinity for CD300lf between the resting and rising conformations generated from the same sample (S16 Fig). However, it is still somewhat speculative. We have added the explanation in Discussion (Line #366) and rephrased the sentence as below (Line #386).

“In consequence, the resting P domain conformation is more accessible to the cellular receptor CD300lf than the rising P domain conformation, causing greater viral infectivity.”

7) l. 218 'The flexible N-terminals formed various interactions with each other between adjacent S domains at the twofold, threefold, pseudo-threefold, and fivefold axes (S8 Appendix B-F)'
This is a secondary point in the paper, but S8 Fig. is a very nice recap of the N-terminal arm's differing positions in Caliciviridae (same subunit vs domain-swapped). The authors should point out that the 5-fold (molecule A) vs 3-fold (i.e. quasi-6-fold, molecule B) positions of the arms are determinants of quasi-equivalence between pentamers (5 'A' arms) and hexamers (3 'B' arms), with the 'C' arms always more disordered.

Authors’ response: Thank you for your suggestion. We have added sentences below in Discussion (Line #272).

“A new feature found in the structure of MNoV is that the N-terminal arm (NTA) in the A monomer extends to the adjacent A monomer, forming a complex of the fivefold axis (S11E Fig). On the other hand, the NTA in the B monomer extends to the adjacent C monomer, forming a complex of the threefold axis (S11F Fig), though the NTA in C monomer is more disordered. Eventually, these NTAs contribute to form the stable icosahedral inner shell.”

8) Minor corrections/clarifications

a) l. 148 'homology' shoud be 'sequence identity'

Authors’ response: We have corrected it as suggested (Line #185).

b) l. 390-392 'two days after infection. MNV was precipitated using ultracentrifugation with a 30% sucrose cushion'
'diluted with DMEM without any additional supplements and precipitated again to remove CsCl.'
Replace 'precipitated' with 'pelleted'.

Authors’ response: We have corrected them as suggested (Line #459 and #461).

c) S2 Table define 'Ri' 'Re'

Authors’ response: To avoid confusion, they have been replaced with symbols and the full notes are attached in the bottom of the table (S2 Table).

d) Fig. 1 '(red arrows in A and C)' ... '(red arrows in B and D)' are almost invisible at printed size (especially A and C)

Authors’ response: Thank you for your suggestion. We have changed the red arrows to black arrowheads to make them clear.

e) S2 Fig. '(B) The cells' please indicate 'HEK293T/CD300lf cells'. In general label the figures so that it is immediately clear which of the two cell types was used.

Authors’ response: We have corrected it as suggested.

f) l. 429 '(C) The early genome replication in each cell incubated for 0, 2, 4, 6, 8, 10, and 12 hours post-infection.' Please describe the protocol. I could not find it in the methods. Presumably cells were pelleted and lysed to separate intracellular from extracellular RNA?

Authors’ response: We have added the description below in Materials and Methods (Line 500).

“RNA was extracted using the same procedure as above and used for qRT-PCR.”

g) l. 771 'MNV-S7 pretreated with PBS(-)-EDTA (pH 8) produced slower propagation of the virus than those pretreated with DMEM, though the difference was small compared to that of MNV-1 (Fig 1E).' All points are actually within error bars. The PBS(-)-EDTA (pH 8) curve is systematically below though, so an appropriate test (Mann-Whitney rank test ?) may be helpful here.

Authors’ response: Thank you for your suggestion. We have included the values of the Mann-Whitney rank test in the legend of new S6 Fig.

h) l. 815 'Asn49 and Asp52 of each C monomer form a charged interaction as shown by the dotted lines'. None of the interactions shown are actually charge-charge (at most one charged residue involved). Please change all 'charged' to 'polar' or 'likely hydrogen bond'.

Authors’ response: Thank you for your suggestion. We have changed all ‘charged’ to ‘polar’ as suggested in text.

Reviewer #3: There are points that should be addressed by the authors before publication. It has been described by others that Ca2+ improves the binding affinity of MNV P-domains for CD300lf by about one order of magnitude, with bile acid, i.e. GCDCA adding another factor of two (Nelson et al., PNAS 2018). The respective crystal structure has well defined binding sites for Ca2+. The authors of that paper discuss the possibility that binding fo MNV to CD300lf is linked to conformational changes of the A’-B’ and the E’-F’ loops, which exist in an open and closed conformation. From this, two questions arise immediately. First, have the present experiments been performed in the absence or in the presence of GCDCA? Second, could the improvement of cell attachment of the resting conformation be the consequence of a combined effect, i.e. „loop conformation“ plus „overall conformation“?

Authors’ response: Thank you for your suggestion. First, we have not performed the binding experiment of rCD300lf in the absence or in the presence of GCDCA. Second, we did not observe a conformational change of the loops in the P2 subdomain when rCD300lf bound, because the map resolution was quite low. However, we had several interesting results, when we compared the cryo-EM model of the P domain having the resting conformation with the previously reported crystallographic model (3QLE) expectedly been in the rising conformation (Taube et al. 2010). The cryo-EM model showed the closed state of the A-B and the E-F loops, which were resemble to the crystal model of 3QLE. However, the orientation of the C-D loop, which functions to connect the paired P domain, was completely different. It may suggest an allosteric effect in the P domain like GCDCA. We have added the explanation in Discussion (from Line #234) with a new S10 Fig.

There is no information about the concentrations of bivalent metal ions, except that binding behavior changes upon addition of EDTA. It would be very helpful to have that information somewhere in the Materials & Methods section.

Authors’ response: In this study, the concentration of bivalent metal ions was not controlled. From our observations, we concluded that the dynamic rotation of the P domain conformation is triggered only by pH, and bivalent metal ions function to stabilize the resting conformation at lower pH. The metal ions would be supplied from DMEM. We have added the explanation below in Discussion (Line #340).

“At present, the metal ions that contribute to domain binding have not been identified, so that the dynamic P domain rotation cannot be controlled by changing their concentrations. To fully understand this mechanism, it is necessary to identify the localization of all metal ions that directly couple the P and S domains must be determined in the future.”
